# DNA methylation–mediated *Rbpjk* suppression protects against fracture nonunion caused by systemic inflammation

**DOI:** 10.1172/JCI168558

**Published:** 2024-02-01

**Authors:** Ding Xiao, Liang Fang, Zhongting Liu, Yonghua He, Jun Ying, Haocheng Qin, Aiwu Lu, Meng Shi, Tiandao Li, Bo Zhang, Jianjun Guan, Cuicui Wang, Yousef Abu-Amer, Jie Shen

**Affiliations:** 1 Department of Orthopaedic Surgery, School of Medicine, Washington University, St. Louis, Missouri, USA.; 2 The Second Xiangya Hospital, Central South University, Changsha, China.; 3 Department of Mechanical Engineering & Materials Sciences, School of Engineering and; 4 Department of Developmental Biology, Center of Regenerative Medicine, Washington University, St. Louis, Missouri, USA.; 5 Shriners Hospital for Children, St. Louis, Missouri, USA.

**Keywords:** Bone Biology, Stem cells, Bone disease, Epigenetics, Gene therapy

## Abstract

Challenging skeletal repairs are frequently seen in patients experiencing systemic inflammation. To tackle the complexity and heterogeneity of the skeletal repair process, we performed single-cell RNA sequencing and revealed that progenitor cells were one of the major lineages responsive to elevated inflammation and this response adversely affected progenitor differentiation by upregulation of *Rbpjk* in fracture nonunion. We then validated the interplay between inflammation (via constitutive activation of Ikk2, Ikk2ca) and Rbpjk specifically in progenitors by using genetic animal models. Focusing on epigenetic regulation, we identified *Rbpjk* as a direct target of Dnmt3b. Mechanistically, inflammation decreased Dnmt3b expression in progenitor cells, consequently leading to *Rbpjk* upregulation via hypomethylation within its promoter region. We also showed that *Dnmt3b* loss-of-function mice phenotypically recapitulated the fracture repair defects observed in *Ikk2ca*-transgenic mice, whereas *Dnmt3b*-transgenic mice alleviated fracture repair defects induced by *Ikk2ca*. Moreover, *Rbpjk* ablation restored fracture repair in both *Ikk2ca* mice and *Dnmt3b* loss-of-function mice. Altogether, this work elucidates a common mechanism involving a NF-κB/Dnmt3b/Rbpjk axis within the context of inflamed bone regeneration. Building on this mechanistic insight, we applied local treatment with epigenetically modified progenitor cells in a previously established mouse model of inflammation-mediated fracture nonunion and showed a functional restoration of bone regeneration under inflammatory conditions through an increase in progenitor differentiation potential.

## Abstract 2

<p>Epigenetic modification of Rbpjk regulates progenitor cell differentiation and protects against fracture nonunion mediated by systemic inflammation.</p>

## Graphical abstract

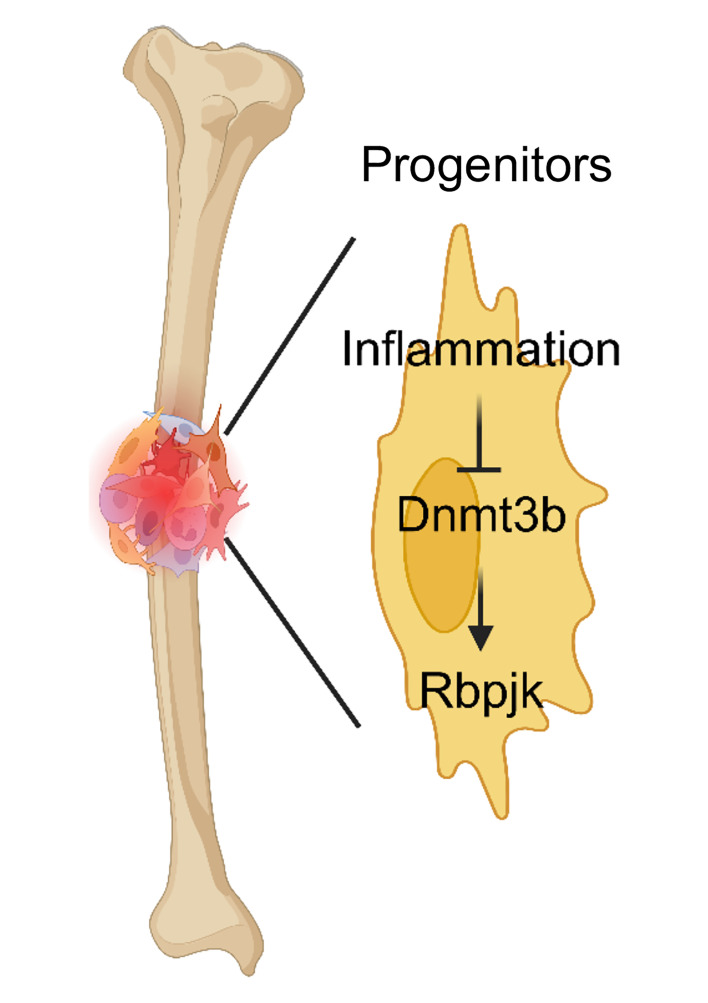

## Introduction

Under most circumstances, bone fractures regenerate with full restoration of tissue morphometry and mechanical strength in the absence of scar formation. Nonetheless, approximately 5% to 10% of fractures globally progress to delayed healing, or nonunion, resulting in prolonged functional disability and increased healthcare expenses (1, 2). The causes of fracture complications in the clinic are multifactorial and include surgery-dependent factors, such as insufficient stabilization and infection, patient-independent factors, such as fracture pattern and severity of trauma, as well as patient-dependent factors, including age, sex, smoking, and other systemic comorbidities (e.g., rheumatoid arthritis [RA], obesity, and metabolic disorders) (2–7). In fact, a majority of patient-dependent factors are closely associated with systemic inflammation (8–10), which places individuals at high risk of experiencing delayed union or nonunion. This underscores the potential detrimental impact of systemic inflammation on fracture healing (11, 12).

Previous investigations in patients and animal models have documented elevated inflammation via the NF-κB pathway leads to reduced expression of chondrogenic and osteogenic genes, thereby adversely affecting progenitor cell differentiation and leading to delay or failure of fracture repair. Notably, intracellular activation of inhibitor of NF-κB kinase subunit β (Ikk2)/NF-κB has been observed to impede chondrogenic and osteogenic differentiation in mice (13, 14). Furthermore, overexpression of TNF-α in transgenic mice has been associated with decreased cortical thickness and inferior bone quality, resulting in a reduction in fracture toughness (15). Our own investigations along with studies from others (15–17) have also demonstrated that systemic inflammation induced by RA disrupts bone regeneration, and in severe cases causes fracture nonunion in mice. In addition to RA models, chronic unbalanced elevation of proinflammatory cytokines in contexts such as aging (18), diabetes (19), and smoking (20) has been identified as the main culprit of skeletal stem/progenitor cell dysfunction. These pathological changes consequently contribute to a reduced regenerative capacity in skeletal systems affected by aging, diabetes, and nicotine exposure. Despite the large amount of evidence that supports the role of inflammation in fracture studies, attempts at biological treatment to mitigate inflammation have not yielded consistent success in clinical outcomes for fracture patients (21), since the precise mechanisms by which pathologic activity of inflammation negatively affects progenitor differentiation and fracture healing have yet to be fully elucidated.

Systemic inflammation has been recognized as a critical modulator of cellular homeostasis via genetic and epigenetic regulation (22–24). In recent years, epigenetics, especially DNA methylation, which is frequently altered in systemic inflammatory conditions, has drawn more attention. For instance, altered DNA methylation landscapes have been identified in various tissues, including synovial fibroblasts, immune cells, and peripheral blood cells in inflammation-related diseases, in particular RA (25–28). More importantly, recent epigenomic studies from fracture patients have revealed differential methylation loci associated with the proliferation and differentiation of human progenitor cells, suggesting that DNA methylation is involved in the fracture repair process (29). Research by our group and others has further established that inflammatory signals (30, 31) disrupt the dynamic regulation of gene expression through DNA methylation, including methylation and demethylation events (32–34), during postnatal degenerative and regenerative processes. Three catalytically active DNA methyltransferases (Dnmts), Dnmt1, Dnmt3a, and Dnmt3b, have been identified in mammalian cells. While Dnmt1 is primarily responsible for maintenance of DNA methylation during cell division (35), the biological functions of the de novo enzymes Dnmt3a and Dnmt3b, which are implicated in the establishment of unique DNA methylation patterns (36–38), remain to be fully elucidated, particularly in the regulation of postnatal tissue regeneration processes. Our earlier work delineated a pivotal role for Dnmt3b in embryonic skeletal development and the maintenance and regeneration of postnatal cartilage homeostasis (39–41). More recently, we provided evidence showing that Dnmt3b was highly expressed in progenitor cells and chondrocytes within fracture callus. Notably, ablation of *Dnmt3b* in either cell type led to a delayed fracture healing, indicating an indispensable role for Dnmt3b in bone regeneration (42, 43). However, the regulation of Dnmts under systemic inflammation is yet to be clarified in the context of fracture repair.

In this study, we performed single-cell RNA sequencing (scRNA-seq) and confirmed the negative impact of systemic inflammation on progenitor cell differentiation through upregulation of Rbpjk during the fracture repair process in RA mice. To gain deeper insights into the underlying cellular and molecular mechanisms, we used transgenic mice with a constitutively active form of Ikk2 (Ikk2ca) in progenitor cells and demonstrated that Rbpjk inhibition mitigated the impairments in progenitor differentiation induced by inflammation, consequently leading to amelioration of the defects observed in fracture repair. Focusing on DNA methylation, we further provided comprehensive in vitro and in vivo evidence that among the Dnmts, Dnmt3b uniquely responded to inflammatory signals. In addition, we revealed Rbpjk as the downstream target that was regulated by Dnmt3b-mediated DNA methylation on its promoter. Building on the insights garnered from this epigenetic mechanism, we engineered progenitor cells with a modified DNA methylation pattern in the *Rbpjk* gene and achieved promising fracture nonunion treatment by applying local cell therapy in RA mice, which represents a promising therapeutic option for delayed fracture and fracture nonunion treatment in the clinic.

## Results

### Systemic inflammation alters progenitor cell differentiation in mice.

**Figure 1:**
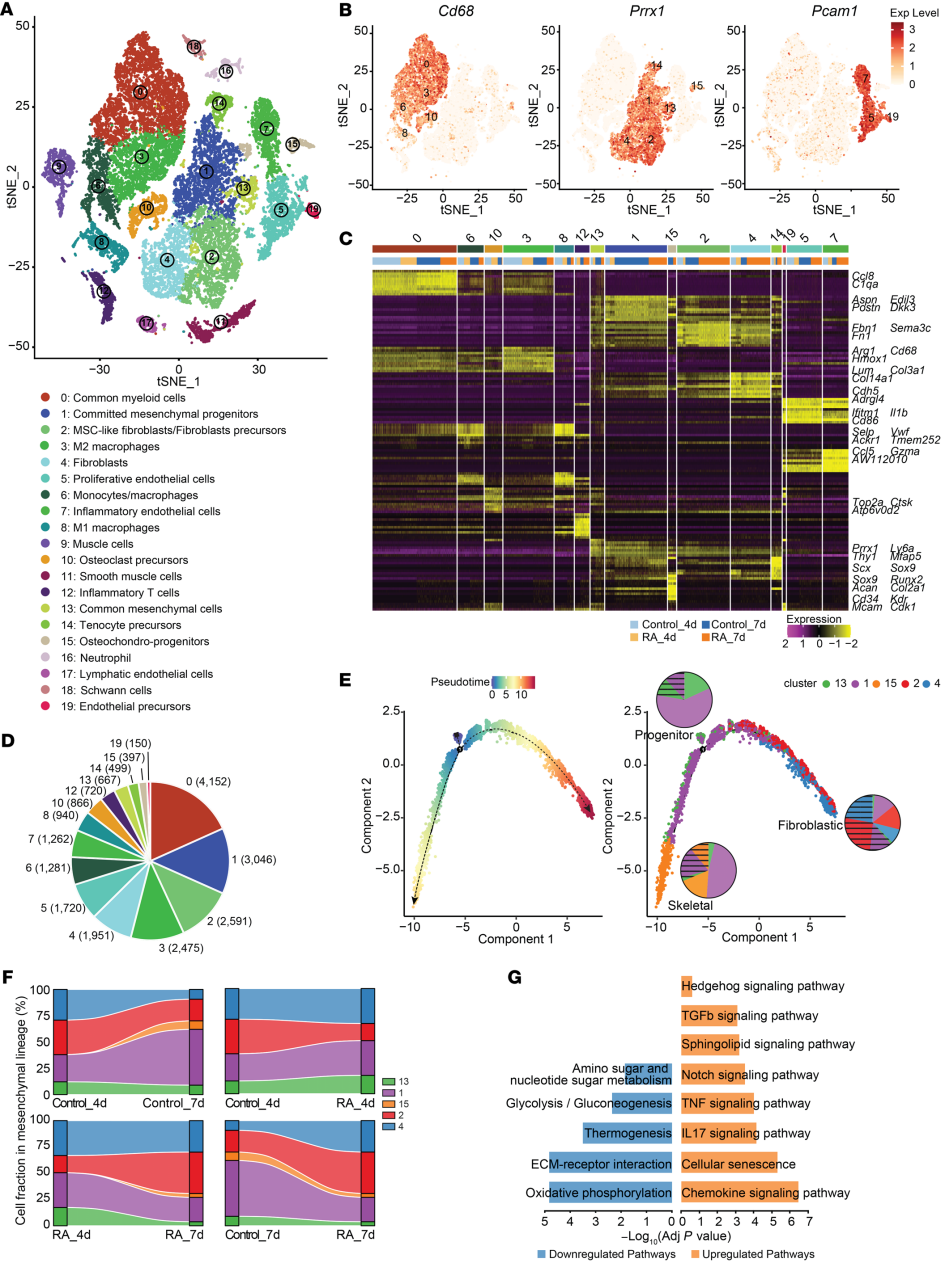
scRNA-seq analysis of fracture callus reveals distinct differentiation trajectories of progenitors in control and RA mice. (**A** and **B**) Twenty cell clusters from control and RA fracture callus at 4 and 7 dpf (*n* = 3). A tSNE projection of 25,467 single-cell transcriptomes, annotated post hoc and colored by clustering (**A**), or by key cell type–specific markers (**B**). (**C**) Cluster signature genes. Expression of top differentially expressed genes (rows) scaled across the cells (columns) in each cluster (color bar on top corresponds to color scheme in **A**), grouped by lineage and conditions (group identity indicated at the bottom). Key genes of some clusters are highlighted on the right. (**D**) Number of cells in each cluster. Color scheme as in **A**. (**E**) Differentiation trajectory of mesenchymal lineage cells constructed by Monocle and colored by pseudotime order (left) and Seurat clusters (right). Cell fractions of each cluster of different conditions (control, unshaded; RA, shaded) shown by pie charts. (**F**) Pair-wise comparison of cellular compositions of mesenchymal subset by conditions (Control_4d vs. Control_7d; RA_4d vs. RA_7d; Control_4d vs. RA_4d; Control_7d vs. RA_7d), visualized by alluvial plots. (**G**) The enriched KEGG pathway analysis of genes unregulated and downregulated in RA fracture callus compared with those in controls.

Fracture repair is a well-organized multistage process that requires the coordinated participation of multiple cell types. Although many studies have documented that systemic inflammation negatively impacts fracture repair, the precise cellular and molecular changes caused by systemic inflammation remain unclear in fracture callus. To address this knowledge gap, we employed a previously established model of inflammation-mediated fracture nonunion (RA mice) (17), isolating fracture callus tissue for subsequent scRNA-seq. To ensure integrity of the cell population and minimize unnecessary contamination of original bone cells and bone marrow cells, we scraped callus tissue along the periosteal surface followed by enzymatic digestion to release cells localized in fracture callus (Supplemental Figure 1; supplemental material available online with this article; https://doi.org/10.1172/JCI168558DS1). Our scRNA-seq analysis, employing an unbiased clustering approach, revealed 20 distinct cellular clusters representing various lineages and potential transitional states (Figure 1, A–D, and Supplemental Figure 2A). Specifically, these clusters encompassed 3 major populations: (a) 6 myeloid lineage subsets characterized by the expression of myeloid marker *Cd68* (Figure 1B and Supplemental Figure 3A), (b) 6 mesenchymal lineage subsets characterized by the expression of *Prrx1* and stem cell antigen 1 (*Sca1*/*Ly6a*) (Figure 1B and Supplemental Figure 3B), and (c) 3 endothelial lineage subsets characterized by the expression of *Pecam1*/*Cd31*, and pan-endothelial marker VE-cadherin (*Cdh5*) (Figure 1B and Supplemental Figure 3C) (44). In line with the elevated systemic inflammation, we identified higher proliferation of inflammatory endothelial cells (cluster 7) and inflammatory T cells (cluster 12) in RA mice, although inflammatory M1 macrophages were decreased. Conversely, a reduction in antiinflammatory M2 macrophages (cluster 3), which play a critical role in resolving inflammation (45), was observed in RA mice (Supplemental Figure 2B).

We then focused on the population of mesenchymal lineage that plays a key role in forming fracture callus. We observed 5 distinct cell types according to lineage-specific gene signatures, e.g., common mesenchymal cells (cluster 13), committed mesenchymal progenitors (cluster 1), osteochondral progenitors (cluster 15), mesenchymal stromal cell–like (MSC-like) fibroblast/fibroblast precursors (cluster 2), and fibroblasts (cluster 4) (Figure 1B and Supplemental Figure 3, A–C). To evaluate cell proliferation and differentiation dynamics, we inferred differentiation trajectories using Monocle and identified a bifurcated path. Notably, common mesenchymal cells (cluster 13) predominantly occupied one trajectory end, whereas osteochondral progenitors (cluster 15), fibroblast precursors (cluster 2), and fibroblasts (cluster 4) were exclusively localized along the opposing, divergent branches. This distribution likely signifies distinct paths of skeletal and fibroblastic differentiation (Figure 1E). We also observed pseudotemporal differences in the lineage progression. The most primitive progenitor cells (cluster 13) were mainly present at early time points. Cluster 1 cells spanned all 3 branches, exhibiting a continuous differentiation time (distance) relative to the initial point, implicating a transitional, intermediate differentiation state with dual potential branching into skeletal and fibroblastic lineages, accompanied by the molecular profile heterogeneity. In contrast, cluster 15 (osteochondral progenitors) and cluster 4 (fibroblasts) were entirely present toward the terminal points of divergent branches, indicative of multipotent common mesenchymal cells. Importantly, analysis of heterogeneity in the bifurcated branches by examining cell fractions revealed that the cells from control fractures were prone to follow the skeletal differentiation pathway, whereas the cells from RA fractures exhibited a greater proportion within the fibroblastic trajectory. Moreover, within the mesenchymal population, examination of cell fractions highlighted a significant increase in cells associated with the skeletal lineage in control fracture callus from 4 to 7 days post fracture (dpf). Conversely, fibroblastic lineage cells became predominant at 7 dpf in RA fracture callus (Figure 1F). Kyoto Encyclopedia of Genes and Genomes (KEGG) analysis of differentially expressed genes within the mesenchymal population revealed enrichment of distinct pathways in RA callus as compared with control mice (Figure 1G). Alongside the anticipated inflammation-related pathways consistent with elevated inflammation in RA fractures, analysis of upregulated genes revealed enrichment for several classic developmental pathways, including Notch, TGF-β, and Hedgehog signaling. Notably, the Notch pathway was the top ranked, with its transcriptional factor *Rbpjk* showing the highest upregulation in uncommitted progenitor cells (cluster 13) (Supplemental Figure 3D), suggesting its potential role in dysregulating mesenchymal differentiation under RA conditions. Interestingly, downregulated gene–enriched pathways were associated with cellular metabolism, thermogenesis, and ECM-receptor interactions, all of which reflect their reduced bone regeneration function in RA mice. Our findings at both the cellular and molecular level suggest that systemic inflammation induces changes in early lineage differentiation from mesenchymal progenitors, consequently impacting subsequent events of bone regeneration.

### Intrinsic inflammation impairs progenitor homeostasis and fracture repair.

**Figure 2:**
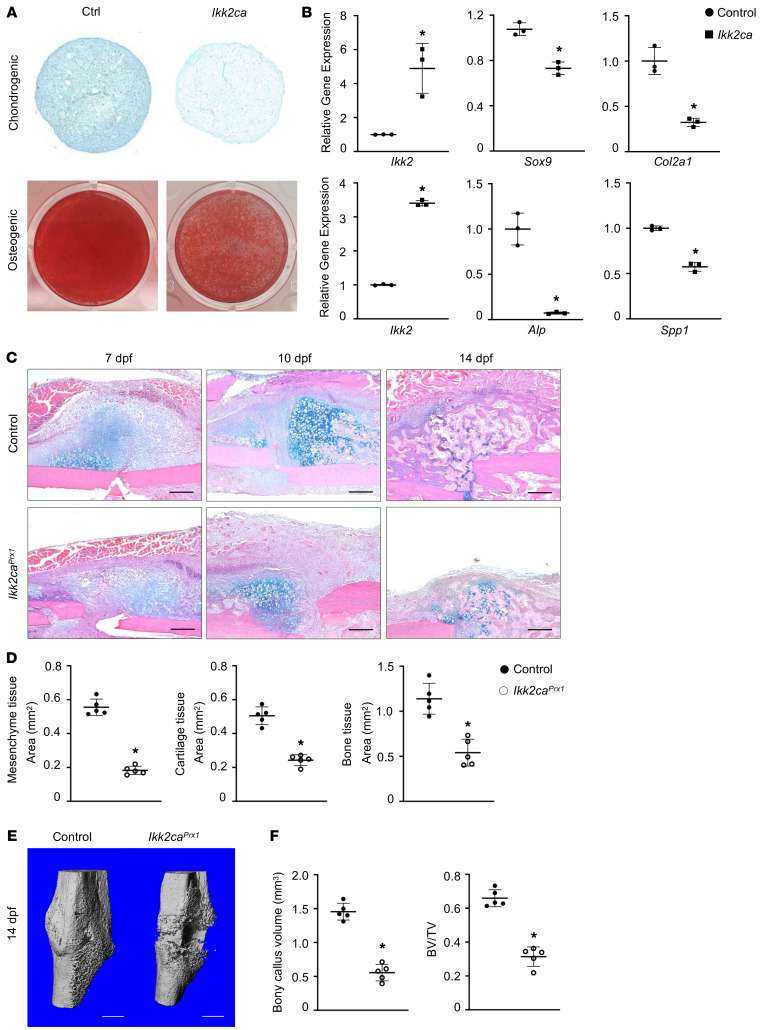
Constitutive activation of *Ikk2* impairs PPC homeostasis and fracture repair. Chondrogenic pellet and osteogenic differentiation assays were performed within *Ikk2ca^fl/+^* PPCs following Ad-GFP (control) or Ad-Cre (*Ikk2ca*) transfection. (**A**) Alcian blue and alizarin red staining of chondrogenic pellet sections and osteogenic cultures on day 28 and day 21, respectively (*n* = 3). (**B**) Real-time qPCR analyses of gene expression for *Ikk2*, *Sox9*, *Col2a1*, *Alp*, and *Spp1* in control or *Ikk2ca* PPCs (*n* = 3). (**C**) ABH/OG staining of fracture callus sections from *Ikk2ca^fl/+^* (control) and *Prx1Cre^ERT2^;Ikk2ca^fl/+^* (*Ikk2ca^Prx1^*) mice at 7, 10, and 14 dpf (*n* = 5). Scale bars: 100 mm. (**D**) Histomorphometric quantifications of mesenchyme, cartilage, and bone areas based on ABH/OG staining (*n* = 5). (**E**) Micro-CT reconstruction of mineralized bony calluses from *Ikk2ca^fl/+^* and *Ikk2ca^Prx1^* mice at 14 dpf (*n* = 5). Scale bars: 0.5 mm. (**F**) Bony callus volume and BV/TV measured on micro-CT assessment of *Ikk2ca^fl/+^* and *Ikk2ca^Prx1^* fractures at 14 dpf (*n* = 5). Data presented as mean ± SD. *P < 0.05 by 2-tailed Student’s *t* test.

In addition to RA fracture callus, we also observed elevated inflammation in the fracture callus isolated from aging mice and high-fat diet–induced obese mice, as reflected by increased *Il1b* and *Tnfa* expression (Supplemental Figure 4). This observation underscores a broader implication of inflammation in the reparative processes of fractures across diverse pathological conditions. It therefore inspired our investigation into the underlying cellular and molecular mechanisms that govern regenerative dysfunction mediated by systemic inflammation. Given that the progenitor lineage was one of the most affected populations in the context of RA callus, we chose to concentrate on periosteal progenitor cells (PPCs), one of the major progenitors contributing to endochondral ossification and fracture callus formation (46). We isolated PPCs from adult (3-month-old) wild-type C57BL/6J mice (Supplemental Figure 5A) and treated them with 1 ng/mL IL-1β. PPCs were indeed responsive to exogenous inflammatory stimuli, as evidenced by increased intrinsic levels of multiple inflammatory factors, including *Ikk2*, *Il1b*, *Il6*, and *Tnfa* (Supplemental Figure 5B). We also induced PPCs with intrinsic inflammation to mimic the persistent state of systemic inflammation caused by *Ikk2ca*. Not surprisingly, proteoglycan production and mineral deposition were reduced in *Ikk2ca* PPCs, as illustrated by the decreased Alcian blue and alizarin red staining during chondrogenesis and osteogenesis, respectively (Figure 2A). Furthermore, key genes associated with chondrogenesis and osteogenesis were also significantly reduced by *Ikk2ca* in PPCs (Figure 2B). Together, these in vitro findings suggest PPCs are responsive to inflammatory signals and elevated intrinsic cellular inflammation induced by *Ikk2ca* detrimentally influences the differentiation of PPCs.

Considering the complexity and heterogeneity of fracture callus, we employed genetic mouse models to provide a more focused understanding of the precise mechanism in progenitor cells. We generated *Prx1Cre^ERT2^;Ikk2ca^fl/+^* (*Ikk2ca^Prx1^*) mice to specifically activate intrinsic inflammatory responses in progenitors. To ascertain the cell lineages targeted by *Prx1Cre^ERT2^* during fracture repair, we examined tibia fractures of *Prx1Cre^ERT2^;Ai9^fl/+^* mice at 7 dpf. Consistent with prior research (47, 48), the *Prx1*-expressing osteochondral progenitors gave rise to all skeletally related cells within fracture callus, in particular the external cartilaginous and bony callus along the periosteal surface (Supplemental Figure 6A), which likely originated from PPCs (47, 49). Following the same strategy as in lineage tracing, we administered tamoxifen 2 weeks prior to creating tibia fractures in *Ikk2ca^Prx1^* and littermate controls (*Ikk2ca^fl/+^*) at 3 months of age. Gene expression analysis revealed an approximately 4- to 6-fold increase in *Ikk2*, *Il1b*, and *Tnfa* expression in 5 dpf *Ikk2ca^Prx1^* fracture callus (Supplemental Figure 6B). Histological evaluations of fracture repair were performed using Alcian blue/hematoxylin/Orange-G (ABH/OG) staining of fracture callus sections at 7, 10, and 14 dpf (Figure 2C). Control mice exhibited normal fracture healing processes, with robust cartilaginous and bony callus formation. In contrast, *Ikk2ca^Prx1^* mice developed impaired healing, with a diminished cartilage template and newly formed woven bone. This observation was confirmed by quantitative histomorphometry at 10 and 14 dpf (Figure 2D). Notably, there appeared to be less mesenchymal tissue initially in *Ikk2ca^Prx1^* fractures; however, it remained at the fracture site by 10 dpf, suggesting compromised progenitor differentiation potential (Figure 2C). Micro-CT imaging of mineralized calluses from control fractures displayed a nearly complete bridging of bony callus by 14 dpf. In contrast, *Ikk2ca^Prx1^* fractures presented with an apparent radiolucent gap between broken cortices, with reduced bony callus formation observed externally on both ends (Figure 2E). Reconstruction of micro-CT data confirmed a significantly reduced volume of newly formed bony callus and a lower bone volume–to–total volume ratio (BV/TV) in *Ikk2ca^Prx1^* fractures (Figure 2F). Furthermore, TRAP staining indicated reduced fracture callus remodeling, evident from decreased osteoclast surface per bone surface (Oc.S/BS) in *Ikk2ca^Prx1^* fractures (Supplemental Figure 7). Collectively, these data strongly suggest that *Ikk2ca* expression disrupts the fracture repair process by impeding progenitor cell differentiation.

### Rbpjk inhibition attenuates Ikk2ca-induced progenitor differentiation and fracture repair defects.

**Figure 3:**
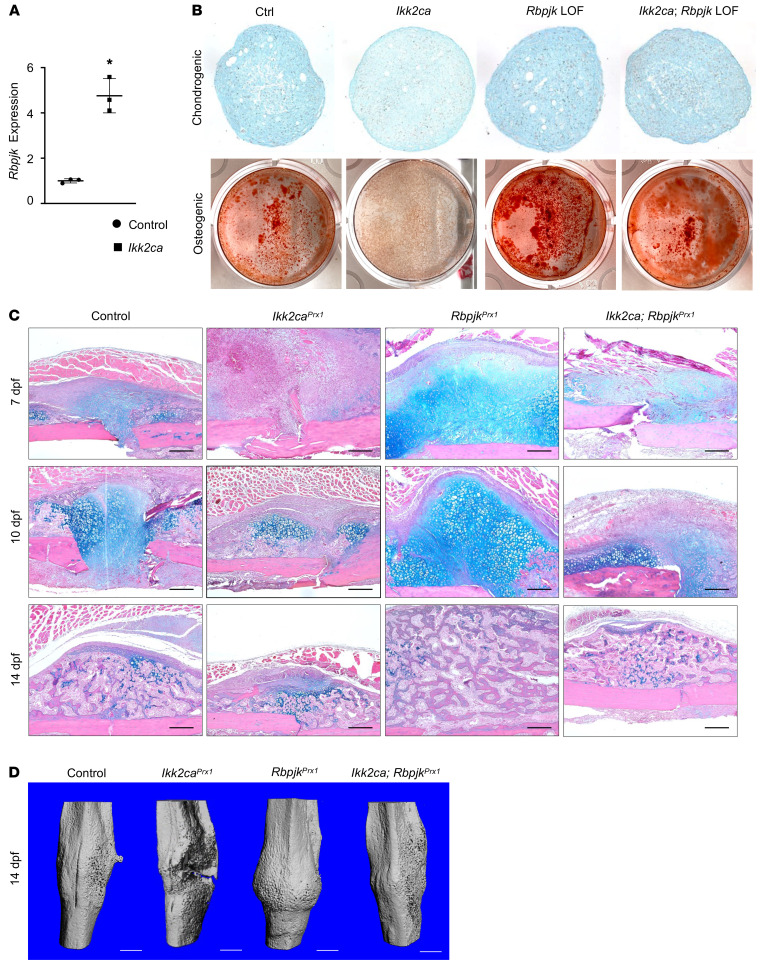
*Rbpjk* inhibition attenuates *Ikk2ca*-induced progenitor differentiation and fracture repair defects. (**A**) Real-time qPCR analyses for *Rbpjk* gene expression within *Ikk2ca^fl/+^* PPCs following Ad-GFP (control) or Ad-Cre (*Ikk2ca*) transfection (*n* = 3). (**B**) Chondrogenic pellet and osteogenic differentiation assays were performed within control and *Ikk2ca* PPCs in the presence or absence of Lenti-sh*Rbpjk* (*Rbpjk* LOF) viral infection. Alcian blue and alizarin red staining of chondrogenic pellet sections and osteogenic cultures on day 28 and day 21, respectively (*n* = 3). (**C**) ABH/OG staining of fracture callus sections from *Ikk2ca^fl/+^* (control), *Prx1Cre^ERT2^;Ikk2ca^fl/+^* (*Ikk2ca^Prx1^*), *Prx1Cre^ERT2^;Rbpjk^fl/fl^* (*Rbpjk^Prx1^*), and *Prx1Cre^ERT2^;Ikk2ca^fl/+^;Rbpjk^fl/fl^* (*Ikk2ca;Rbpjk^Prx1^*) mice at 7, 10, and 14 dpf (*n* = 5). Scale bars: 100 mm. (**D**) Micro-CT reconstruction of mineralized bony calluses from control, *Ikk2ca^Prx1^*, *Rbpjk^Prx1^*, and *Ikk2ca;Rbpjk^Prx1^* mice at 14 dpf (*n* = 5). Scale bars: 0.5 mm. Data expressed as mean ± SD. *P < 0.05 determined by 2-tailed Student’s *t* test for comparisons between 2 groups.

After identifying the Rbpjk-mediated Notch pathway as the most regulated pathway in progenitor lineages under systemic inflammatory conditions, and observing a nearly 5-fold increase in *Rbpjk* expression in PPCs due to intrinsic cellular inflammation induced by *Ikk2ca* (Figure 3A), our subsequent investigation aimed to establish whether the upregulation of *Rbpjk* directly contributed to the progenitor differentiation disruption and fracture defects induced by *Ikk2ca*. As expected, we found that progenitor differentiation deficiencies caused by *Ikk2ca* were mitigated by *Rbpjk* ablation, as indicated by the restoration of chondrogenic and osteogenic potential in *Ikk2ca;Rbpjk* loss-of-function (LOF) PPCs (Figure 3B).

To further explore the possibility of alleviating impaired fracture healing in *Ikk2ca^Prx1^* mice through *Rbpjk* ablation, we generated *Prx1Cre^ERT2^;Ikk2ca^fl/+^*;*Rbpjk^fl/fl^* double-mutant mice (*Ikk2ca;Rbpjk^Prx1^*, *Ikk2ca;Rbpjk* LOF) by crossing *Prx1Cre^ERT2^;Ikk2ca^fl/+^* with *Rbpjk^fl/fl^* mice. In the context of *Ikk2ca^Prx1^* fractures, impaired fracture healing was consistently observed (Figure 3C). In accordance with previous findings achieved by a pharmacological approach (42, 50), genetic deletion of *Rbpjk* in progenitors resulted in enhanced endochondral ossification, evident in quantitative histomorphometry (Supplemental Figure 8). Importantly, the impaired fracture healing induced by *Ikk2ca* was restored by *Rbpjk* ablation in progenitor cells. Quantitative assessments of cartilaginous and bony callus areas revealed no significant difference between the *Ikk2ca;Rbpjk* LOF mutant and control fractures (Figure 3C and Supplemental Figure 8). Moreover, micro-CT images and quantitative measures of newly formed bone further illustrated that *Rbpjk* ablation resulted in enhanced endochondral ossification and restoration of fracture repair defects, in particular the unification of broken cortices in *Ikk2ca^Prx1^* mice (Figure 3D and Supplemental Figure 9). Additionally, under the *Ikk2ca^Prx1^* background, *Rbpjk* LOF restored fracture callus remodeling at 14 dpf (Supplemental Figure 10). Collectively, the findings from both in vitro and in vivo experiments suggest that intrinsic cellular inflammation driven by *Ikk2ca* upregulates *Rbpjk* in progenitor cells that, in turn, results in compromised progenitor differentiation and delayed fracture repair.

### Inflammation leads to Rbpjk upregulation via DNA methylation changes in progenitor cells.

**Figure 4:**
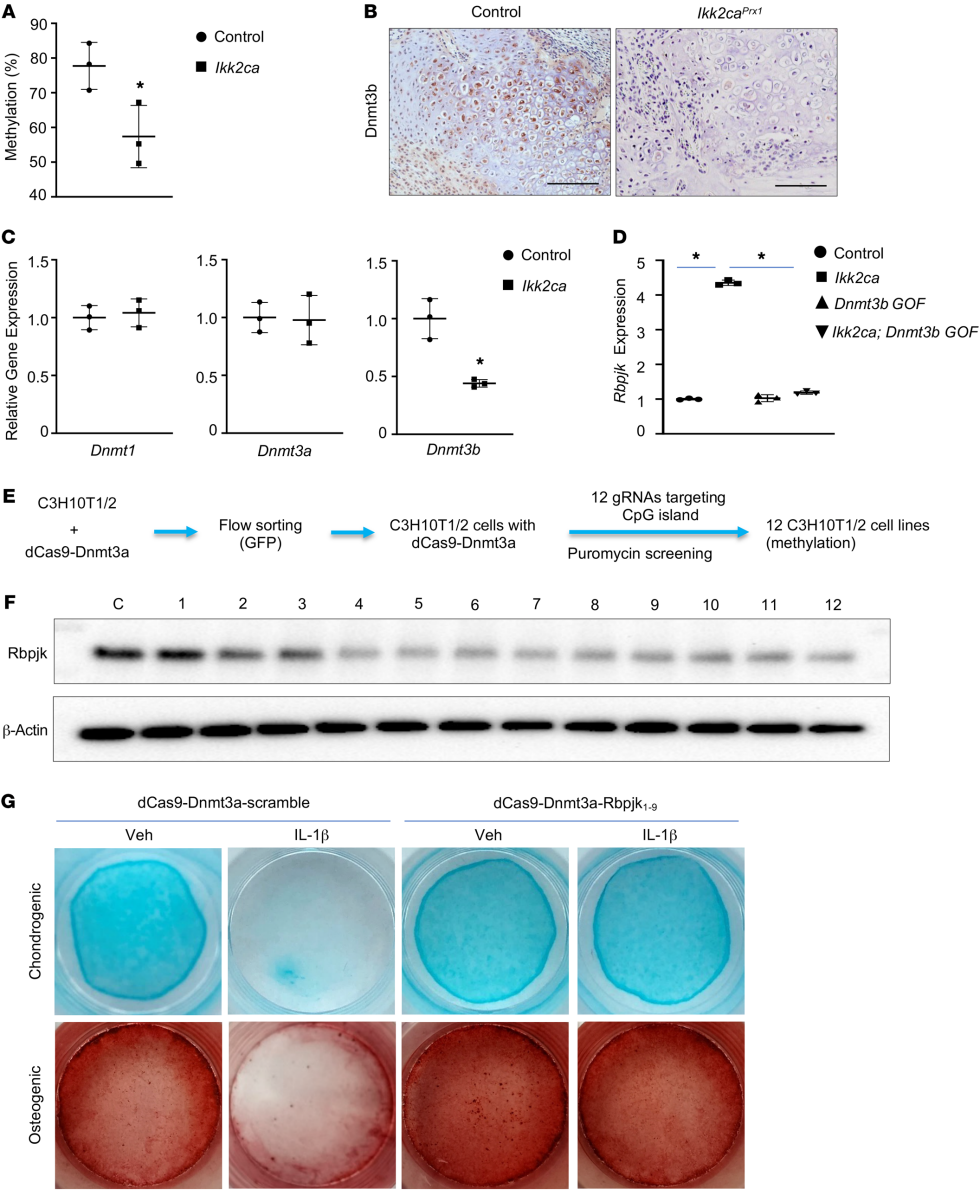
Inflammation induces *Rbpjk* expression through Dnmt3b-mediated DNA methylation reduction. (**A**) Methylation qPCR for the *Rbpjk* promoter region in *Ikk2ca^fl/+^* PPCs following Ad-GFP (control) or Ad-Cre (*Ikk2ca*) transfection (*n* = 3). (**B**) IHC for Dnmt3b on callus sections from *Ikk2ca^fl/+^* (control) and *Prx1Cre^ERT2^;Ikk2ca^fl/+^* (*Ikk2ca^Prx1^*) mice at 10 dpf (*n* = 5). Scale bars: 100 mm. (**C**) Real-time qPCR analyses of gene expression for *Dnmt1*, *Dnmt3a*, and *Dnmt3b* in control and *Ikk2ca* PPCs (*n* = 3). (**D**) Real-time qPCR analyses for *Rbpjk* within control and *Ikk2ca* PPCs in the presence or absence of Lenti-*Dnmt3b* (*Dnmt3b* GOF) viral infection (*n* = 3). (**E**) Schematic representing the selection of C3H10T1/2 cell lines modified by the dCas9-Dnmt3a epigenetic editing system specifically targeting CpG islands of the *Rbpjk* gene. (**F**) Western blot analyses for Rbpjk in protein extracts from dCas9-Dnmt3a-scramble gRNA (control) and 12 individual dCas9-Dnmt3a-Rbpjk gRNA-engineered C3H10T1/2 cell lines (*n* = 3). Lane C: dCas9-Dnmt3a-scramble gRNA. Lanes 1–10: dCas9-Dnmt3a-Rbpjk_1-1-10_ gRNAs targeting CpG island 1 of the *Rbpjk* gene. Lanes 11 and 12: dCas9-Dnmt3a-Rbpjk_2-1-2_ gRNAs targeting CpG island 2 of the *Rbpjk* gene. (**G**) Chondrogenic pellet and osteogenic differentiation assays were performed within dCas9-Dnmt3a-scramble (control) and dCas9-Dnmt3a-Rbpjk_1-9_ C3H10T1/2 cell lines in the presence or absence of IL-1β. Alcian blue and alizarin red staining of chondrogenic and osteogenic cultures on day 28 and day 21, respectively (*n* = 3). Data expressed as mean ± SD. *P < 0.05 determined by 2-tailed Student’s *t* test (**A** and **C**) or by 2-way ANOVA followed by Tukey’s test (**D**).

To understand the mechanism by which *Ikk2ca* regulates Rbpjk, our primary focus was on investigating the epigenetic regulation, particularly DNA methylation. Epigenetic DNA modifications have recently emerged as an important hallmark of inflammatory diseases (28, 51, 52), and there is evidence linking DNA methylation changes to fracture-related complications in patients (29). Our data underscored a reduction of approximately 25% in DNA methylation within the *Rbpjk* promoter region in *Ikk2ca* PPCs, therefore reinforcing our hypothesis (Figure 4A). With a specific emphasis on DNA methylation, we initiated an examination of Dnmt expression patterns under *Ikk2ca* during fracture repair. Intriguingly, we observed a significant decrease in *Dnmt3b* expression in 5 dpf *Ikk2ca^Prx1^* callus, while *Dnmt1* and *Dnmt3a* remained unaffected (Supplemental Figure 11). Immunohistochemistry (IHC) further validated diminished Dnmt3b staining in *Ikk2ca^Prx1^* callus, in contrast with its robust expression in control callus among progenitors and chondrocytes (Figure 4B). Consistent with in vivo observations, both *Ikk2ca* (Figure 4C) and IL-1β treatment (Supplemental Figure 12) exclusively suppressed expression of *Dnmt3b* in PPCs, with *Dnmt1* and *Dnmt3a* unaffected. More importantly, CpG islands, known as Dnmt3b binding sites, were identified in the *Rbpjk* promoter and gene body, and ChIP assays validated Dnmt3b’s interaction with these binding sites in PPCs (Supplemental Figure 13). Reintroducing *Dnmt3b* into *Ikk2ca* PPCs led to downregulation of *Rbpjk* to levels comparable to controls (Figure 4D). Altogether, these findings strongly support the notion that Dnmt3b is the sole Dnmt responsive to inflammatory signals, and the upregulation of *Rbpjk* due to DNA methylation changes is likely attributable to Dnmt3b suppression.

To further confirm the role of DNA methylation in Rbpjk regulation, we utilized the CRISPR/dCas9 editing system to manipulate local DNA methylation within the *Rbpjk* gene. Compared with other methylation editing systems (53, 54), the dCas9/Dnmt3a system exhibited higher efficiency and precision in engineering DNA methylation (55). dCas9-Dnmt3a with 1 single guide RNA (gRNA) could induce an approximately 30% increase in DNA methylation within a 150-bp region (55). Accordingly, we designed 12 specific gRNAs to cover both CpG islands in the *Rbpjk* gene, including 1.5 kb in the promoter and 300 bp in the gene body (Supplemental Figure 14A). Lentiviral delivery of dCas9-Dnmt3a was used to transduce C3H10T1/2 murine progenitor cells. Specific gRNAs, validated by next-generation sequencing (Table 1), were employed to guide the methylation activity in the *Rbpjk* gene. Following selection, 12 individual C3H10T1/2 cell lines were obtained for downstream in vitro functional assays (Figure 4E). Western blot analyses confirmed reduced Rbpjk protein expression in each cell line (except cell line 1, Figure 4F). Conversely, we employed the dCas9/Tet1 editing system in conjunction with 12 specific gRNAs to reduce local DNA methylation in *Rbpjk* (Supplemental Figure 14B), and demonstrated that reduced DNA methylation resulted in upregulation of Rbpjk in cells. These findings provide compelling evidence supporting 2 CpG islands as the key epigenetic elements in the regulation of Rbpjk expression in progenitor cells.

Next, to establish the connection between inflammation and DNA methylation in the *Rbpjk* gene, we explored whether maintenance of local DNA methylation in the *Rbpjk* gene could restore progenitor cell differentiation under inflammatory conditions. We selected the dCas9-Dnmt3a-Rbpjk_1-9_ cell line, which exhibited the most reduced Rbpjk protein expression among the 12 cell lines (Table 2) for in vitro cell differentiation assays. Bisulfite sequencing analysis validated an approximately 1.5-fold increase in DNA methylation within our targeted promoter region in dCas9-Dnmt3a-Rbpjk_1-9_ cells (Supplemental Data File 1). As a result, no upregulation of *Rbpjk* or its direct downstream target *Hey1* was observed in dCas9-Dnmt3a-Rbpjk_1-9_ cells compared to control (dCas9-Dnmt3a-scramble) cells upon IL-1β stimulation (Supplemental Figure 15), indicating effective epigenetic editing of the *Rbpjk* gene. Importantly, unlike the diminished cell differentiation observed in control cells, the differentiation capacity remained unaltered in dCas9-Dnmt3a-Rbpjk_1-9_ cells under inflammatory conditions, as illustrated by similar intensity of Alcian blue and alizarin red staining between control and IL-1β–treated cells (Figure 4G). Gene expression analyses during differentiation mirrored the pattern observed in the stained differentiation cultures. The markers associated with chondrogenic (*Sox9*, *Col2a1*, and *Acan*) and osteogenic (*Sp7*, *Runx2*, and *Alp*) differentiation were markedly reduced by IL-1β in control cells (Supplemental Figure 15), suggesting impaired cell differentiation. However, there were no significant differences in gene expression between vehicle- and IL-1β–treated dCas9-Dnmt3a-Rbpjk_1-9_ cells during differentiation. In fact, both groups exhibited comparable or slightly enhanced expression levels compared with vehicle-treated control cells (Supplemental Figure 15), indicating that the downregulation of *Rbpjk* via epigenetic modification effectively restored cell differentiation capacity.

### Dnmt3b deficiency–mediated Rbpjk upregulation causes impaired fracture repair under inflammatory conditions.

**Figure 5:**
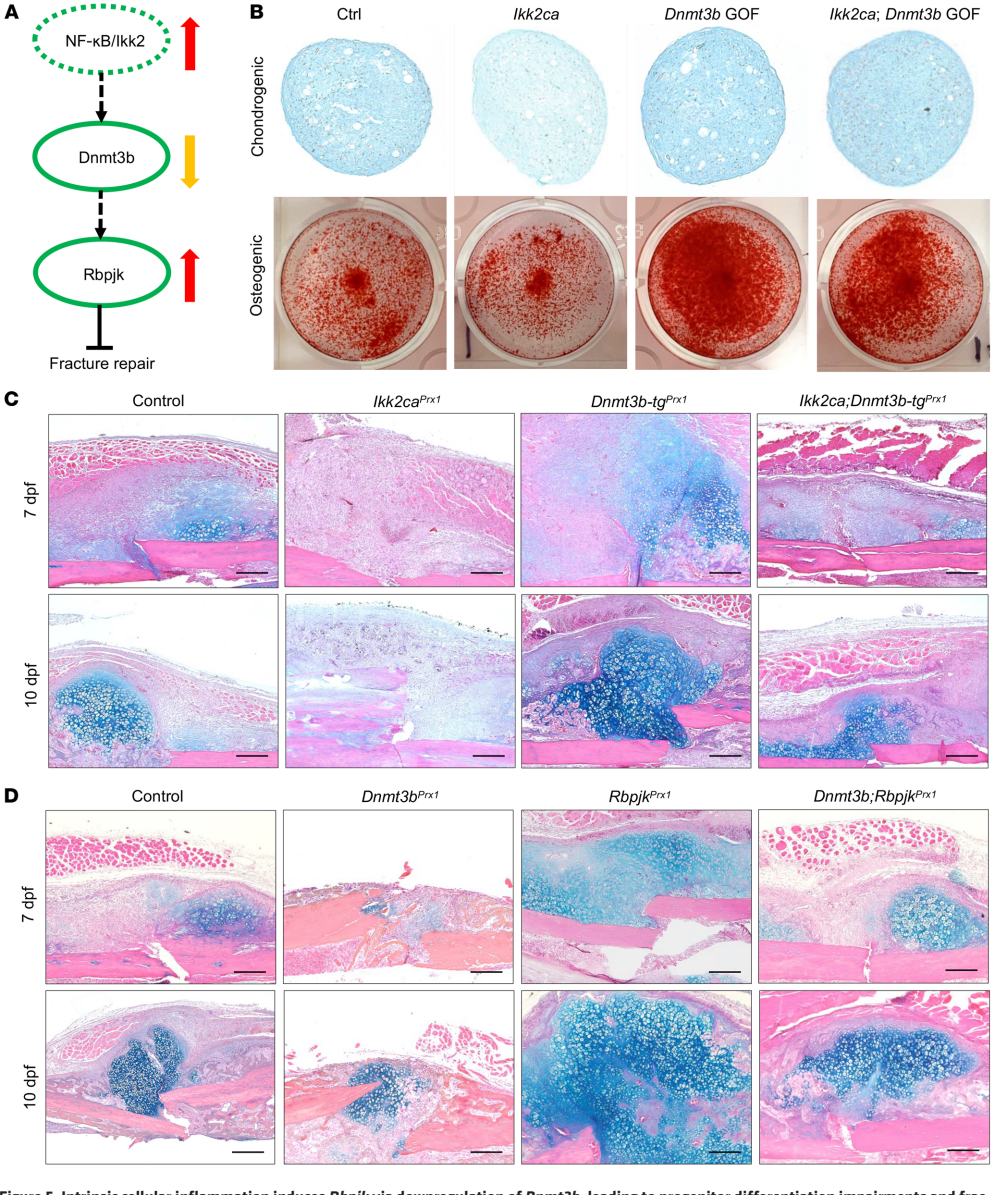
Intrinsic cellular inflammation induces *Rbpjk* via downregulation of *Dnmt3b*, leading to progenitor differentiation impairments and fracture repair defects. (**A**) Schematic demonstration of previously established NF-κB/Dnmt3b/Rbpjk axis in regulating fracture repair. Dashed line: Relationship established by in vitro experiments. Solid line: Relationship established in mice. Dashed circle: Role of the gene established in vitro. Solid circle: Role of the gene established in mice. (**B**) Chondrogenic pellet and osteogenic differentiation assays were performed within control and *Ikk2ca* PPCs in the presence or absence of Lenti-*Dnmt3b* (*Dnmt3b* GOF) viral infection. Alcian blue and alizarin red staining of chondrogenic pellet sections and osteogenic cultures on day 28 and day 21, respectively (*n* = 3). (**C**) ABH/OG staining of fracture callus sections from *Ikk2ca^fl/+^* (control), *Prx1Cre^ERT2^;Ikk2ca^fl/+^* (*Ikk2ca^Prx1^*), *Prx1Cre^ERT2^;Rosa-rtTA^fl/+^;Dnmt3b*-Tg (*Dnmt3b-tg^Prx1^*), and *Prx1Cre^ERT2^;Ikk2ca^fl/+^;Rosa-rtTA^fl/+^;Dnmt3b*-Tg (*Ikk2ca;Dnmt3b-tg^Prx1^*) mice at 7 and 10 dpf (*n* = 5). Scale bars: 100 mm. (**D**) ABH/OG staining of fracture callus sections from *Dnmt3b^fl/fl^* (control), *Prx1Cre^ERT2^;Dnmt3b^fl/fl^* (*Dnmt3b^Prx1^*), *Prx1Cre^ERT2^;Rbpjk^fl/fl^* (*Rbpjk^Prx1^*), and *Prx1Cre^ERT2^; Dnmt3b^fl/fl^;Rbpjk^fl/fl^* (*Dnmt3b;Rbpjk^Prx1^*) mice at 7 and 10 dpf (*n* = 5). Scale bars: 100 mm.

In our previous investigations utilizing murine models, we established impaired endochondral ossification and fracture repair as a result of *Dnmt3b* ablation (42) and *Rbpjk* deletion (47) in progenitor cells (Figure 5A). Encouraged by the in vitro evidence showing interactions among *Ikk2*, *Dnmt3b*, and *Rbpjk* in progenitor cells, we were motivated to validate the Ikk2/Dnmt3b/Rbpjk axis and explore its role in regulating progenitor cell homeostasis by using genetic mouse models. As Dnmt3b emerged as the exclusive Dnmt responsive to both systemic and intrinsic inflammation, and considering that *Dnmt3b* LOF similar to *Ikk2ca* hindered progenitor cell differentiation and fracture healing in mice, we examined whether *Dnmt3b* overexpression could compensate for *Ikk2ca*-induced cellular defects. *Ikk2ca^fl/+^* PPCs were isolated and infected with Ad-Cre and/or Lenti-*Dnmt3b* to overexpress *Ikk2* and/or *Dnmt3b*, followed by chondrogenic and osteogenic assays (Figure 5B). *Ikk2ca* consistently attenuated chondrogenesis and osteogenesis in PPCs, as evidenced by diminished Alcian blue and alizarin red staining. Notably, *Dnmt3b* overexpression mitigated progenitor differentiation defects induced by *Ikk2ca*, evident in the restored chondrogenesis and osteogenesis that resembled those in control cells. To further validate those observations in vivo, we generated tissue-specific and inducible *Prx1Cre^ERT2^;Ikk2ca^fl/+^;Rosa-rtTA ^fl/+^;tetO-Dnmt3b* (*Ikk2ca;Dnmt3b-tg^Prx1^*, *Ikk2ca;Dnmt3b* gain-of-function [GOF]) mice to determine whether *Dnmt3b* overexpression could protect against the inflammation-induced fracture repair defects driven by *Ikk2ca* in progenitor cells. Prior to fractures in 3-month-old mice, tamoxifen and doxycycline were administered to activate *Ikk2* and *Dnmt3b*, respectively, in progenitor cells (Supplemental Figure 16). Histological assessments showed that *Ikk2ca^Prx1^* mice consistently exhibited impaired fracture healing (Figure 5C). *Dnmt3b* GOF (*Dnmt3b-tg^Prx1^*) mice displayed enhanced fracture repair, reflected by more robust cartilaginous callus formation. More importantly, *Ikk2ca;Dnmt3b* GOF mice exhibited bone repair process in a timely manner comparable to that observed in control fractures, suggesting that *Dnmt3b* GOF protected against fracture repair defects induced by *Ikk2ca* in progenitors (Figure 5C). These histological observations were further supported by quantitative measures of fracture callus composition at 7 and 10 dpf for mesenchyme and cartilage area, respectively (Supplemental Figure 17). In addition, accelerated mineralization of bony callus in *Dnmt3b* GOF fractures and timely complete bone bridging in *Ikk2ca;Dnmt3b* GOF fractures were further demonstrated by micro-CT images and quantification of bony callus volume and BV/TV ratio (Supplemental Figure 18).

Regarding the interplay between *Dnmt3b* and *Rbpjk*, we examined whether *Rbpjk* LOF could mitigate the cellular defects mediated by *Dnmt3b* LOF. *Dnmt3b^fl/fl^;Rbpjk^fl/fl^* PPCs were isolated and infected with Ad-Cre to induce *Dnmt3b* and *Rbpjk* knockdown, followed by chondrogenic and osteogenic assays. Similar to *Ikk2ca*, *Dnmt3b* LOF led to reduced chondrogenesis and osteogenesis in PPCs. However, under *Dnmt3b* LOF conditions, *Rbpjk* LOF restored PPC differentiation capacity, as evidenced by improved Alcian blue and alizarin red staining in *Dnmt3b;Rbpjk* LOF cells (Supplemental Figure 19). We then generated *Prx1Cre^ERT2^;Dnmt3b^fl/fl^*;*Rbpjk^fl/fl^* mice (*Dnmt3b;Rbpjk^Prx1^*, *Dnmt3b;Rbpjk* LOF) and observed a restored cartilaginous callus formation in *Dnmt3b;Rbpjk* LOF mice along with increased mesenchyme and cartilage tissue at 7 and 10 dpf, respectively (Figure 5D and Supplemental Figure 20). Micro-CT analysis further confirmed a timely and robust bony callus formation, characterized by improved bony callus volume and BV/TV ratio in 14 dpf *Dnmt3b;Rbpjk* LOF callus compared with *Dnmt3b* LOF callus (Supplemental Figure 21). Together with in vitro mechanistic evidence, these findings imply that the suppression of *Dnmt3b* leading to *Rbpjk* upregulation, at least in part, contributes to the impaired bone healing induced by *Ikk2ca* in progenitors.

### Rbpjk inhibition by genetic and epigenetic engineering approaches rescues fracture nonunion in RA mice.

**Figure 6:**
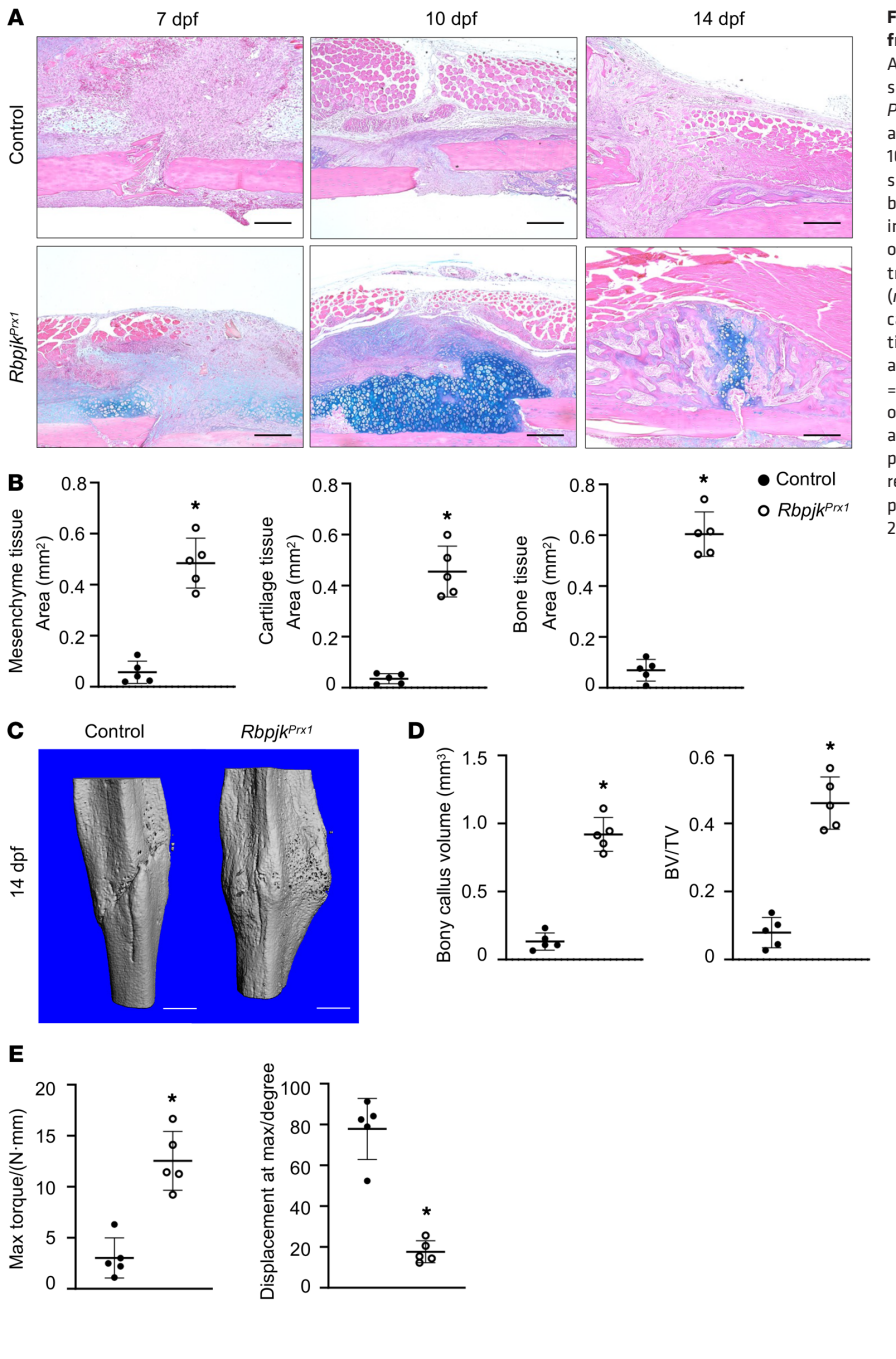
*Rbpjk* ablation abrogates fracture nonunion of RA mice. (**A**) ABH/OG staining of fracture callus sections from *Rbpjk^fl/fl^* (control) and *Prx1Cre^ERT2^;Rbpjk^fl/fl^* (*Rbpjk^Prx1^*) RA mice at 7, 10, and 14 dpf (*n* = 5). Scale bars: 100 mm. (**B**) Histomorphometric measures of mesenchyme, cartilage, and bone areas based on ABH/OG staining (*n* = 5). (**C**) Micro-CT assessment of mineralized bony calluses of control and *Rbpjk^Prx1^* RA mice at 14 dpf (*n* = 5). Scale bars: 0.5 mm. (**D**) Bony callus volume and BV/TV quantifications on micro-CT analyses of control and *Rbpjk^Prx1^* RA fractures at 14 dpf (*n* = 5). (**E**) Biomechanical torsion testing of control and *Rbpjk^Prx1^* RA fractures at 28 dpf. Maximum torque and displacement at maximum torque were recorded during testing (*n* = 5). Data presented as mean ± SD. *P < 0.05 by 2-tailed Student’s *t* test.

Given that both Ikk2ca-mediated inflammation and Dnmt3b broadly influence the entire genome, Rbpjk is a more specific downstream target capable of regulating progenitor cell differentiation and bone repair. To assess the feasibility of Rbpjk inhibition as a therapeutic approach, we employed a well-established RA fracture nonunion model to simulate systemic inflammation–induced fracture complications. We utilized *Prx1Cre^ERT2^;Rbpjk^fl/fl^* (*Rbpjk^Prx1^*, *Rbpjk* LOF) along with its littermate controls (*Rbpjk^fl/fl^*), administering K/BxN arthritogenic serum during the fracture repair process. As expected, *Rbpjk^fl/fl^* RA mice exhibited a lack of bony union and failure of fracture repair, as revealed by the absence of apparent callus formation with persistence of undifferentiated tissue (Figure 6, A and B), unbridged broken cortices with minimal mineralized callus (Figure 6, C and D), and compromised biomechanical properties (Figure 6E). In contrast, under the same induction of systemic inflammation, *Rbpjk^Prx1^* mice displayed a comparatively normal repair process. Histological assessment showed an increase in cartilaginous and bony callus formation, occurring in a timely fashion (Figure 6, A and B). This in turn resulted in significantly greater bony callus and complete fracture unification in *Rbpjk^Prx1^* RA mice, as confirmed in micro-CT analyses (Figure 6, C and D). Additionally, the remodeling of fracture callus was accelerated in *Rbpjk^Prx1^* RA mice, as evidenced by increased Oc.S/BS in 14 dpf callus (Supplemental Figure 22). Ultimately, biomechanical properties, serving as a definitive indicator of fracture repair outcome, were evaluated by torsion testing on the repaired tibia at 28 dpf. Compared with *Rbpjk^fl/fl^* RA mice, *Rbpjk^Prx1^* RA mice exhibited a significant increase in maximum torque (4.3-fold increase), with a decreased displacement angle, indicating markedly improved mechanical competence (Figure 6E).

In addition to genetically ablating *Rbpjk* in progenitor cells, we also explored a translational approach: cell therapy in the RA fracture nonunion model. To this end, we employed a previously documented scaffold made of polycaprolactone (PCL), a US FDA–approved biodegradable polymer for tissue engineering applications (56). To provide a proof of concept and determine the feasibility, we fabricated tissue containing simultaneously electrospinning PCL fibers and electrospraying the adipose-derived GFP^+^ progenitors (86) (Supplemental Figure 23A). Scaffold architecture and cell distribution were characterized by scanning electron microscopy and confocal fluorescence microscopy, respectively. These analyses revealed that PCL fibers closely mimicked collagen morphology in tissues, offering sufficient micropores for cell migration (Supplemental Figure 23B). Moreover, the GFP^+^ progenitor cells were evenly distributed in the scaffolds, evident from GFP fluorescence (Supplemental Figure 23C). More importantly, upon implantation at the fracture site, the GFP^+^ progenitor cells within the scaffolds exhibited the capacity to differentiate into both cartilaginous and bony callus (Supplemental Figure 23D), suggesting that the scaffolds themselves did not impede fracture repair, but instead facilitated localized cell therapy.

**Figure 7:**
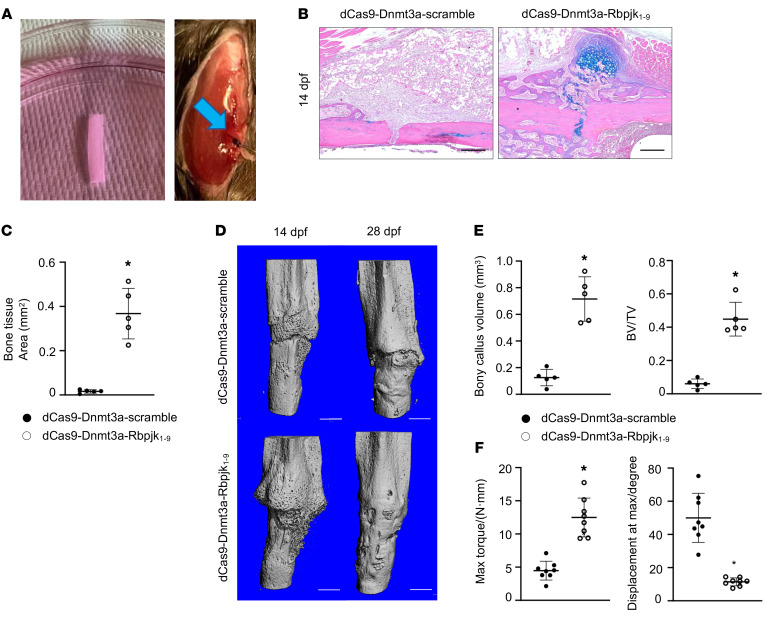
Rbpjk inhibition by epigenetic modification using CRISPR/dCas9/Dnmt3a editing system rescues fracture nonunion in RA mice. (**A**) PCL scaffold with dCas9-Dnmt3a–engineered C3H10T1/2 cells (blue arrow) was applied to the fractured bone in RA mice. (**B**) ABH/OG staining of callus sections from 14-dpf wild-type RA fractures grafted with PCL scaffolds that were fabricated with dCas9-Dnmt3a-scramble (control) or dCas9-Dnmt3a-Rbpjk_1-9_ C3H10T1/2 cells (*Rbpjk* epigenetically modified) (*n* = 5). Scale bars: 100 mm. (**C**) Histomorphometric quantifications of bone area based on ABH/OG staining (*n* = 5). (**D**) Micro-CT analyses of newly formed bony callus from 14-dpf wild-type RA fractures grafted with PCL scaffolds that were fabricated with control or *Rbpjk* epigenetically modified C3H10T1/2 cells (*n* = 5). Scale bars: 0.5 mm. (**E**) Bony callus volume and BV/TV measures of 14-dpf wild-type RA fractures grafted with PCL scaffolds fabricated with control or *Rbpjk* epigenetically modified C3H10T1/2 cells (*n* = 5). (**F**) Biomechanical torsion testing of 28-dpf wild-type RA fractures grafted with PCL scaffolds fabricated with control or *Rbpjk* epigenetically modified C3H10T1/2 cells. Maximum torque and displacement at maximum torque were recorded during testing (*n* = 8). Data presented as mean ± SD. *P < 0.05 by 2-tailed Student’s *t* test.

Encouraged by the in vivo bioactivity of PCL scaffold–delivered progenitors, we moved on to employ the scaffolds loaded with epigenetically modified dCas9-Dnmt3a-Rbpjk_1-9_ C3H10T1/2 cells (3 × 10^5^ cells/cm^3^) in RA fractures. As previously established, immediately following fractures, a 2-mm scaffold (~200 μm thick) was wrapped around the fractured bone of RA mice (Figure 7A). Histological and micro-CT evaluations revealed that RA fractures treated with PCL scaffolds encapsulating dCas9-Dnmt3a-scramble cells were characterized by the persistence of mesenchymal tissue and the absence of a unified rigid structure (Figure 7, B–E). In contrast, PCL scaffolds loaded with dCas9-Dnmt3a-Rbpjk_1-9_ C3H10T1/2 cells induced a regenerative response. This was evidenced by the emergence of new vessel formation at 10 dpf (Supplemental Figure 24) as well as enhanced bony callus formation, fracture unification, and restored callus remodeling at 14 dpf (Figure 7, B–D, and Supplemental Figure 25) under RA conditions. Notably, the maximum bone strength in RA mice treated with dCas9-Dnmt3a-Rbpjk_1-9_ C3H10T1/2 cells was significantly restored by 28 dpf, coincident with reduced degrees of displacement during torsion testing (Figure 7F). Hence, these findings provided robust evidence that PCL scaffold–mediated localized delivery of progenitors, wherein *Rbpjk* is inhibited via epigenetic modifications, represents a potent therapeutic strategy for addressing delayed fracture union or nonunion under inflammatory conditions.

## Discussion

Despite various factors contributing to delayed fracture union and nonunion, systemic inflammation has emerged as a prominent concern due to its increasing prevalence in conditions such as aging (18), obesity/diabetes (19), smoking (6), and other inflammatory diseases (11, 12). Consistent with clinical observations, recent animal studies, including our recently developed RA-associated fracture nonunion model, have consistently demonstrated that systemic inflammation negatively impacts progenitor cell proliferation and differentiation, leading to compromised fracture healing in rodents. With the efforts to tackle systemic inflammation and its related comorbidities, pharmacological anti-cytokine therapies have been developed and are highly effective in treating systemic inflammatory diseases (57); however, their impact on fracture repair in patients remains uncertain. For instance, a study using an anti-TNF antibody in a mouse model demonstrated improved fracture healing (58), while contradictory results were observed in an ankylosing spondylitis patient cohort treated with a TNF-α inhibitor (59). This underscores the complexity of the effects of anti-cytokine treatments on fracture repair outcomes. Therefore, there is an urgent need to uncover the underlying mechanisms and develop mechanism-based therapies to address delayed fracture healing and nonunion under conditions of systemic inflammation.

In this regard, the current study was driven by a mechanistic approach aimed at shedding light on the role of systemic inflammation during fracture repair and identifying potential therapeutic targets for future clinical interventions. Given the complexity and heterogeneity of the fracture repair process, we employed scRNA-seq to identify and profile individual cells within fracture callus. This comprehensive analysis aimed to decipher how pathological conditions, particularly systemic inflammation, alter the cellular and molecular programs of skeletal regeneration. In accordance with clinical observations and our previous findings that serum transfer–induced RA mice develop fracture nonunion (17), our scRNA-seq analysis demonstrated an accumulation of inflammatory cells and a reduction in endothelial cells within the RA callus. More importantly, the scRNA-seq data revealed that progenitor cells in RA mice differentiated into fibroblast cells rather than osteochondrally committed cells, suggesting a disruption in progenitor cell proliferation and differentiation due to systemic inflammation. Of particular interest within the progenitor lineages, we identified several distinct pathways that were altered in primitive progenitor cells expressing *Prx1*, among which the Rbpjk-mediated Notch pathway stood out as one of the most upregulated pathways. These findings, coupled with our knowledge that Rbpjk upregulation impairs progenitor differentiation and leads to fracture nonunion (47), prompted us to further investigate the link between inflammation and Rbpjk in progenitor cells by using genetic mouse models. To this end, we selectively activated *Ikk2*, the principal mediator of inflammatory responses, in progenitor cells, which resulted in reduced progenitor cell differentiation potential and impaired fracture healing in mice. Moreover, we demonstrated that Rbpjk is at least one of the critical downstream targets of Ikk2ca in progenitor cells, regulating both progenitor cell differentiation and fracture repair. These findings were consistent with scRNA-seq data from the systemic inflammation (RA) scenario. Notably, single-allele *Ikk2* activation in progenitor cells resulted in an approximately 5-fold increase in inflammatory cytokines (*Il1b* and *Tnfa*) in fracture callus, which was comparable to the increase observed in patient sera with systemic inflammatory conditions (e.g., aging, obesity, diabetes, and RA) ranging from 2- to more than 40-fold (60–64). This relevance of pathological inflammatory conditions in *Ikk2ca* animal model enhances the translational potential of our mechanistic findings. Although sex differences may need further investigation, our experience and pilot experiments suggest no apparent differences in fracture healing and inflammatory responses between male and female mice.

To delve deeper into the regulation of Rbpjk by Ikk2ca in progenitor cells, we focused on epigenetic regulation, specifically DNA methylation. Growing evidence indicates that aberrant epigenetic modifications, including DNA methylation, are associated with various systemic inflammatory diseases, reflecting to some extent the downstream effects of systemic inflammatory cues. Epigenetic variations have recently been linked to bone-related diseases, including fractures, influencing the functional capacity of progenitors, osteogenic differentiation, angiogenesis, and bone remodeling (29, 65–68). Changes in DNA methylation have been identified in bone marrow MSCs of myeloma bone disease, particularly in genes involved in osteogenic differentiation, leading to their abnormal expression and impaired bone formation (67). Our group’s work has demonstrated the requisite role of Dnmt3b-mediated DNA methylation signatures in both progenitors (42) and differentiated chondrocytes (43) for normal fracture repair. Furthermore, we observed a reduction in DNA methylation in the *Rbpjk* promoter region in *Ikk2ca* progenitor cells. This suggests that Ikk2ca induces Rbpjk expression at least partially through inhibition of Dnmts. We demonstrated in this study that Dnmt3b, rather than Dnmt1 or Dnmt3a, was responsive to inflammatory signals and reduced under inflammatory conditions. This reduction was observed not only in progenitor cells treated with inflammatory cytokines in vitro, but also in the callus of mice with RA. Overexpression of *Ikk2* in progenitors resulted in a similar pattern of *Dnmt3b* reduction in vitro and in vivo. The *Dnmt3b*-deficient mice phenotypically resembled activation of *Ikk2* in progenitors regarding fracture repair. Furthermore, we demonstrated that overexpression of *Dnmt3b* alleviated fracture repair defects induced by *Ikk2* activation. Mechanistically, ChIP assay and dCas9-mediated local DNA methylation modification assay confirmed the direct interaction of Dnmt3b with its binding site, maintaining DNA methylation and suppressing *Rbpjk* gene expression. Conversely, loss of DNA methylation due to Dnmt3b reduction resulted in *Rbpjk* upregulation in progenitor cells. In addition to in vitro experiments, we also provided compelling in vivo evidence demonstrating that *Rbpjk* ablation restored fracture repair in *Dnmt3b*-deficient mice as well as in RA and *Ikk2ca* mice. Collectively, these findings established a NF-κB/Dnmt3b/Rbpjk axis and highlighted its role in maintaining progenitor function during skeletal repair under conditions of systemic inflammation.

While it is acknowledged that Notch signaling components, including Rbpjk and NOTCH intracellular domain (NICD), can also be genetically regulated by inflammatory stimuli via the TAK1-mediated noncanonical pathway in various cells (69, 70), our enthusiasm lies in the epigenetic regulation of Rbpjk. Epigenetic variations are more individual-specific and likely reflect personal disease profile, as opposed to the variability of gene expression levels. Recent studies involving longitudinal personalized DNA methylomics in humans have suggested that DNA methylation profiles are associated with systemic conditions, providing insights distinct from changes in gene expression linked to acute health conditions (71, 72). Hence, exploring epigenetic biomarkers and developing epigenetics-based therapeutic interventions, particularly for disorders associated with systemic inflammation, holds great promise. As a proof of concept, our study demonstrated, to the best of our knowledge for the first time, the feasibility and regenerative potential of epigenetics-based therapy in treating fracture nonunion using a clinically relevant RA model. To enhance the effectiveness of epigenetically modified cell therapy, we engineered a PCL scaffold for maintenance and local delivery of progenitors at fracture sites. PCL scaffold, an FDA-approved biodegradable material, has been successfully utilized for bone regeneration in preclinical studies (17, 73, 74). Notably, our fabricated scaffolds closely mimicked periosteum tissue morphology and strength, presenting a fibrous and porous structure. The optimization of the epigenetic editing system and cell numbers will be a focus of future studies. Overall, our work suggests that the initial identification of epigenetic biomarkers followed by the development of locus-specific epigenetic modifiers could represent an important therapeutic option, enabling personalized medicine to address delayed union and nonunion.

In summary, this study has revealed a fundamental mechanism by which systemic inflammation, mediated by the NF-κB pathway, triggers a cascade of events that ultimately leads to epigenetic aberrations, thus negatively impacting fracture repair. We have elucidated an NF-κB/Dnmt3b/Rbpjk axis that highlights the interplay between inflammation and epigenetics in the regulation of progenitor cell regenerative potential. Our compelling findings establish that Dnmt3b-mediated processes are key modulators of progenitor cell responsiveness to inflammatory cues during bone regeneration. The functional decline of progenitor cells and the resulting defects in fracture repair can be restored by epigenetic modification of DNA methylation specifically at the *Rbpjk* promoter. These findings provide strong suggestions for the development of epigenetics-based therapy aimed at addressing delayed bone union and nonunion. Although our study has extensively explored the epigenetic regulation of Rbpjk, particularly owing to the Notch pathway being the most upregulated under systemic inflammation, other significantly altered pathways, such as TGF-β and cellular metabolism, are also worthy of further investigation. The TGF-β pathway has been shown to be associated with aging-related cell senescence and fibrotic tissue formation in the context of fractures (75). Cellular metabolism has also emerged as a critical factor influencing immune cells, osteoblasts, and progenitor cells in the regulation of bone repair processes under pathological conditions (76–78). These pathways could provide avenues for understanding and potentially modulating fracture repair outcomes.

## Methods

### Mice.

Male C57BL/6J wild-type mice purchased from The Jackson Laboratory (stock 000664) were used for the RA fracture model. *Prx1Cre^ERT2^* (79), *Ikk2ca^fl/fl^* (80), *Ai9^fl/fl^* (81), and *Rbpjk^fl/fl^* (82) mice have been previously described and were purchased from The Jackson Laboratory (stock 029211, 008242, 007909, and 034200, respectively). *Dnmt3b^fl/fl^* (37) mice were obtained from the Mutant Mouse Resource and Research Centers (catalog 29887). *Dnmt3b*-transgenic (*Dnmt3b*-Tg) mice were previously generated in the Shen laboratory. Upon recombination, *Dnmt3b* expression is induced with doxycycline (40). Thus, the model provided both cell-specific and temporal regulation of *Dnmt3b* overexpression. *Dnmt3b*-Tg mice were crossed with *Prx1Cre^ERT2^;Rosa-rtTA^fl/+^* mice to generate *Dnmt3b* GOF mice. *Prx1Cre^ERT2^;Ai9^fl/+^*, *Prx1Cre^ERT2^;Ikk2ca^fl/+^*, *Prx1Cre^ERT2^;Rosa-rtTA^fl/+^;Dnmt3b*-Tg, *Prx1Cre^ERT2^;Ikk2ca^fl/+^;Rosa-rtTA^fl/+^;Dnmt3b*-Tg, *Prx1Cre^ERT2^;Rbpjk^fl/fl^*, *Prx1Cre^ERT2^;Dnmt3b^fl/fl^;Rbpjk^fl/fl^*, and *Prx1Cre^ERT2^;Ikk2ca^fl/+^;Rbpjk^fl/fl^* were viable and produced in Mendelian ratios. All mice were fractured at 3 months of age. Two weeks prior to fractures, mice that carry the *Prx1Cre^ERT2^* transgene received tamoxifen (1 mg/10 g body weight) via intraperitoneal (i.p.) injections for 5 consecutive days. Doxycycline (2.5 mg/kg body weight) was delivered i.p. to *Prx1Cre^ERT2^;Rosa-rtTA^fl/+^;Dnmt3b*-Tg and *Prx1Cre^ERT2^;Ikk2ca^fl/+^;Rosa-rtTA^fl/+^;Dnmt3b*-Tg and their littermates 1 day prior to fracture surgery. Doxycycline administration continued twice per week following fractures until mice were sacrificed for downstream analyses. Systemic inflammation was induced via i.p. injection of 100 μL of arthritogenic K/BxN serum on 0 dpf and 3 dpf and maintained by continuous injection every 5 days until the experimental endpoint. Mice receiving PBS served as controls. Bony fractures were created on the right tibiae as previously documented (43).

### scRNA-seq and data processing.

Callus samples were collected by scraping newly formed tissue along the periosteal surface and dissociated in 1 mg/mL Collagenase P (MilliporeSigma, 11213865001) at room temperature for 1 hour. The cell suspension was filtered through a 30-μm strainer to generate a single-cell suspension. Four batches of single-cell libraries were sequenced: callus cells from 4 dpf control fractures (control_4d, *n* = 3), 7 dpf control fractures (control_7d, *n* = 3), 4 dpf RA fractures (RA_4d, *n* = 3), and 7 dpf RA fractures (RA_7d, *n* = 3). Ten thousand cells for each sample were loaded for preparing single-cell mRNA libraries for each group, barcoded, purified using a Chromium Single Cell 3′ kit (v3.1 Chemistry, 10× Genomics Inc), and sequenced using a 2 × 150-bp paired-end configuration on an Illumina NovaSeq platform. The CellRanger pipeline (v7.0.1, https://support.10xgenomics.com/single-cell-gene-expression/software/pipelines/latest/using/what-is-cell-ranger) was used to align reads, extract cell barcode and unique molecular identifier (UMI) counts, and generate feature-barcode matrices, after mapping to the mouse (mm10) reference genome, according to the standard workflow of 10× Genomics.

### scRNA-seq analysis.

Quality control was conducted for each data set. Multiplets or cells with poor quality (UMI < 4,000, UMI > 50,000, features < 2,000, features > 7,000, or >8% mitochondrial UMIs) were excluded. Data set integration, graph-based cell clustering, dimensionality reduction, and data visualization were performed using Seurat v4.3.0 (83). Feature data were normalized using the Seurat NormalizeData function (a global-scaling normalization method) and then scaled with Seurat ScaleData function (a linear transformation) in order to remove unnecessary variations from unwanted sources for downstream analyses. Nonlinear dimensional reduction was performed with t-distributed stochastic embedding (tSNE) and graph-based clustering was achieved by applying the Louvain algorithm. The top 15 statistically significant principal components were chosen empirically to identify the dimensionality of the data set by testing top 10 differentially expressed genes (DEGs) (Wilcox’s method) among clusters. Annotation of cell clusters was performed by examining cell type–specific marker gene expression across clusters. KEGG enrichment analysis was performed by uploading DEGs of interest into Enrichr (84) and overlaying with global signaling pathways from the KEGG database to identify significant pathways. Trajectory analysis of the mesenchymal population was performed using Monocle v2.24.1 (https://cole-trapnell-lab.github.io/monocle-release/). ggplot2 (https://ggplot2.tidyverse.org/) and ggalluvial (https://corybrunson.github.io/ggalluvial/) packages were used to generate data graphs.

### Histological analyses.

The fractured tibiae (*n* = 5; 3 females and 2 males) were collected for histological analyses at 7, 10, and 14 dpf. Following fixation with 10% neutral buffered formalin and decalcification using 14% ethylenediaminetetraacetic acid (EDTA), the fractured tibiae were embedded in paraffin, sectioned at 5 μm, and stained with ABH/OG. The mesenchyme, cartilage, and bone areas were measured using ImageJ software (NIH). Tissues prepared for frozen sections were fixed in 4% paraformaldehyde for 2 hours at 4°C, decalcified with 14% EDTA for 10 days, infiltrated with gradient sucrose for 3 days, embedded with Tissue-TEK OCT medium, and sectioned at a thickness of 10 μm. GFP and tdTomato fluorescence from frozen sections of fracture callus was examined by Zeiss upright fluorescence microscope.

### Micro-CT analyses.

The fractured tibiae (*n* = 5; 3 females and 2 males) were harvested at 14 dpf and assessed by a micro-CT scanner (Viva CT40, Scanco) with the following parameters: 55 kV, 145 μA, and a 300 ms integration time. Three-dimensional images of the entire callus were constructed using Scanco software. Quantifications of the newly formed BV and BV/TV were performed on 600 slices centered on the midpoint of the fracture site, as previously described (43).

### Biomechanical torsion testing.

Due to known biomechanical differences between male and female tibiae, the biomechanical torsion test was performed on male mice. The fractured tibiae (*n* = 5–8) from male mice were collected at 28 dpf and both ends were potted with methacrylate (MMA) bone cement (Lang Dental Manufacturing) and secured in 1.2-cm-long cylinders (6 mm diameter) to keep the fracture site in the middle. After MMA solidified, the bone samples with the cylinders were set up on the torsion machine using the custom LabVIEW (National Instruments) program until failure. The maximum torque and displacement at maximum torque were recorded and processed by a custom MATLAB 2017 program (MathWorks).

### PPC isolation and culture.

Murine PPCs were isolated from periosteum of 3-month-old wild-type or *Ikk2ca^fl/+^* mice as previously described (85). Briefly, after carefully removing soft tissue, both ends of the long bone were capped with 5% agarose. The entire long bone was then placed in 10 mL of 1 mg/mL Collagenase P (MilliporeSigma, 11213865001) for 10 minutes to remove muscle and other soft tissue remnants. The murine PPCs were collected after a 1-hour digestion with 1 mg/mL Collagenase P solution and were seeded in 6-well plates for expansion. The primary PPCs from passages 3–5 were used for experiments. In order to overexpress *Ikk2* in PPCs, Ad-GFP and Ad-Cre (MOI = 50) were used to treat primary murine PPCs for 24 hours followed by 24-hour recovery. After recovery, Lenti-*Dnmt3b* (MOI = 10; Origene, MR225598L4V) and Lenti-sh*Rbpjk* (MOI = 10; Origene, TL512813V) were used to infect adenovirus-treated *Ikk2ca^fl/+^* PPCs to overexpress *Dnmt3b* and knock down *Rbpjk* in cells, respectively.

### Chondrogenic and osteogenic differentiation assays.

PPCs (2 × 10^5^) were centrifuged to form cell pellets and cultured in 15 mL conical tubes under hypoxic conditions for chondrogenic differentiation for up to 28 days. The chondrogenic differentiation medium was composed of DMEM supplemented with 100 nM dexamethasone, 50 mg/mL L-ascorbic acid 2-phosphate, 40 mg/mL L-proline, 100 mg/mL sodium pyruvate, and 10 ng/mL TGF-β3. IL-1β treatment (1 ng/mL) and chondrogenic differentiation medium were changed every 3 days. qPCR was performed 14 days after chondrogenic differentiation induction and Alcian blue staining was performed 28 days after chondrogenic differentiation induction. PPCs (1 × 10^5^) were seeded in 12-well plates under normoxic conditions for osteogenic differentiation for up to 21 days. The osteogenic differentiation medium consisted of DMEM supplemented with 10% FBS, 100 nM dexamethasone, 10 mM sodium β-glycerophosphate, and 50 mg/mL ascorbic acid. IL-1β treatment (1 ng/mL) and osteogenic differentiation medium were changed every 3 days. qPCR was performed 7 days after osteogenic differentiation induction and alizarin red staining was performed 21 days after osteogenic differentiation induction.

### Epigenetic modification of Rbpjk using CRISPR/dCas9 editing system.

Local DNA methylation editing was achieved by using the dCas9/Dnmt3a platform (55). Briefly, C3H10T1/2 cells (ATCC, CCL-226) were transfected with Lenti-dCas9-Dnmt3a (Addgene, 84476). After 3 days of culture, GFP^+^ C3H10T1/2 cells were collected by FACS. gRNAs (Table 2) designed specifically for *Rbpjk* CpG islands were then delivered into C3H10T1/2 cells carrying the dCas9-Dnmt3a cassette to modify local DNA methylation. Twelve individual C3H10T1/2 cell lines were generated by puromycin selection. The genomic DNA was isolated from the epigenetically engineered C3H10T1/2 cells and was processed with a bisulfite conversion kit (QIAGEN, 59104) for subsequent Sanger sequencing to examine the local DNA methylation. The percentage DNA methylation increase within the 300-bp CpG island region was calculated by comparing dCas9-Dnmt3a-Rbpjk_1-9_ cells to dCas9-Dnmt3a-scramble cells based on the Sanger sequencing data. Similarly, the dCas9/Tet1 editing platform (55) was used to reduce local DNA methylation of the *Rbpjk* gene. C3H10T1/2 cells were transfected with Lenti-dCas9-Tet1 (Addgene, 84475). The same gRNAs (Table 2) were used to reduce DNA methylation in specific regions of the *Rbpjk* gene. In total, 12 individual C3H10T1/2 cell lines were generated by puromycin selection.

### Real-time qPCR and Western blot analyses.

Four-millimeter fracture calluses (*n* = 5) were collected from experimental mice and homogenized for RNA extraction using an RNeasy Mini Kit (QIAGEN, 74106). cDNA synthesis and real-time qPCR were performed according to the manufacturers’ instructions. Primer sequences are presented in Table 3. Methylation qPCR for *Rbpjk* was performed according to the manufacturer’s instructions (QIAGEN). Genomic DNA was isolated from primary PPCs and separately digested with methylation-sensitive and methylation-dependent enzymes. The enzyme-digested genomic DNA was then used for qPCR and the primers for *Rbpjk* methylation (CpG Island 106810) were obtained from QIAGEN (EPMM106810). Protein lysates were fractioned by SDS-polyacrylamide gel electrophoresis and examined with antibodies against Rbpjk (1:500; Santa Cruz Biotechnology, sc-271128) and β-actin (1:1000; MilliporeSigma, 2228).

### Statistics.

All data were analyzed using GraphPad Prism and are presented as mean ± SD of at least 3 independent experiments. A 2-tailed Student’s *t* test was used to determine the significance between 2 groups. Two-way analysis of variance (ANOVA) was used to compare among multiple groups, with subsequent pairwise comparisons examined via Tukey’s test. A *P* value of less than 0.05 was considered statistically significant.

### Data availability.

Raw scRNA-seq data can be accessed via the NCBI Gene Expression Omnibus (GEO GSE242836). Values for all data points in graphs can be found in the supplemental Supporting Data Values file.

### Study approval.

All animal studies were performed in accordance with approval of the Committees on Animal Resources at Washington University in St. Louis.

## Author contributions

YA, JG, CW, and JS participated in experimental design. DX, LF, ZL, YH, JY, AL, HQ, MS, TL, BZ, and CW performed the experiments and data analysis. ZL fabricated the PCL scaffold with progenitor cells under the supervision of JG. The YA lab provided K/BxN serum. CW, YA, JG, and JS participated in manuscript writing and revision. CW guided the scRNA-seq design and data analysis. JS guided the experimental approach throughout the duration of the project and finalized the manuscript. The order of co–first authors was assigned based on the experiments conducted.

## Supplementary Material

Supplemental data

Supplemental data set 1

Supporting data values

## Figures and Tables

**Table 3:**
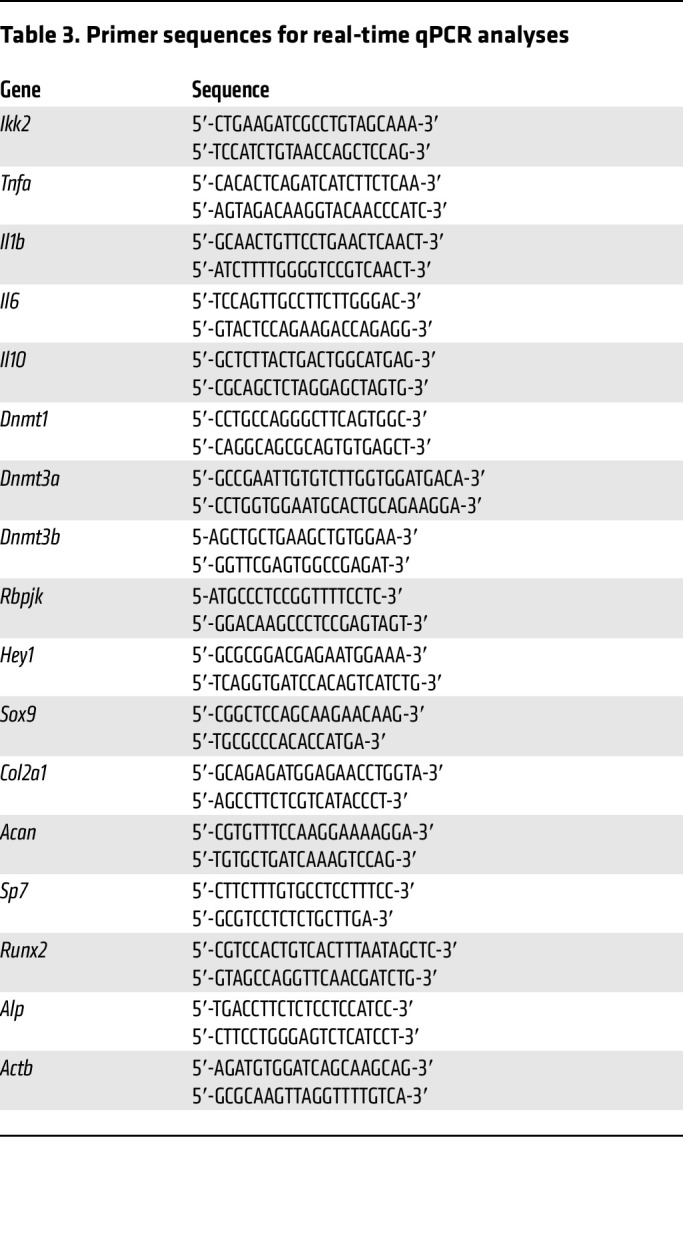
Primer sequences for real-time qPCR analyses

**Table 2:**
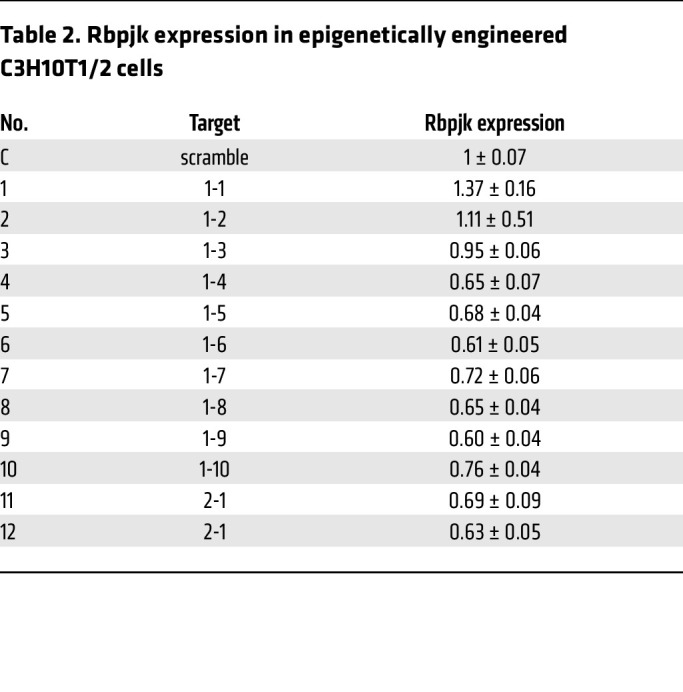
Rbpjk expression in epigenetically engineered C3H10T1/2 cells

**Table 1:**
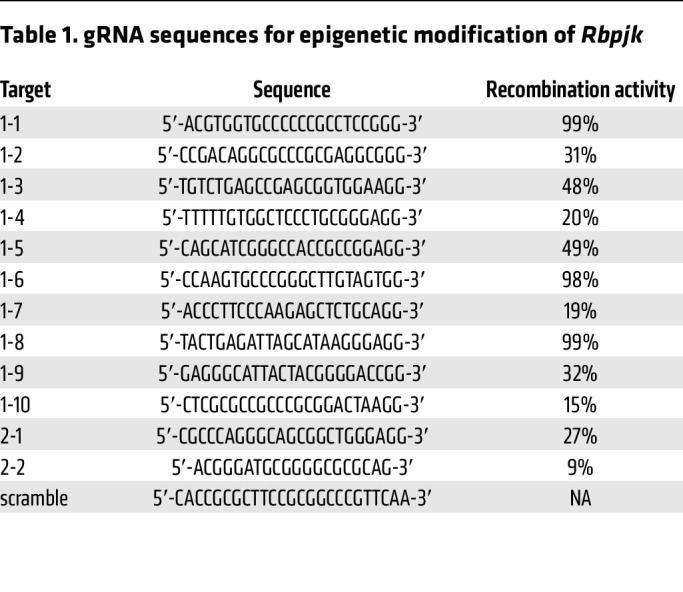
gRNA sequences for epigenetic modification of *Rbpjk*

## Version 1 12/05/2023

In-Press Preview

## Version 2 02/01/2024

Electronic publication

## Version 3 02/02/2024

Color key corrected in Figure 1G
