# Supplementary material for: DNA methylation–mediated Rbpjk suppression protects against fracture nonunion caused by systemic inflammation
Source: J Clin Invest. 2024 Feb 1;134(3):e168558. doi: 10.1172/JCI168558 (PMC10849763; doi:10.1172/JCI168558)
Supplement: Supplemental data [file jci-134-168558-s058.pdf]

Supplemental methods

Flow cytometry analysis. The cultured PPCs (passage 3 to passage 5) were prepared for flow cytometry analysis with fluorochrome conjugated antibodies including CD45 monoclonal antibody (1:200; ThermoFisher, #64-9459-42), CD90.2 monoclonal antibody (1:200; ThermoFisher, #47-0902-82), and Sca1 monoclonal antibody (1:150; ThermoFisher, #45-5981-82).

ChIP assay. PPCs (passage 3 to passage 5, 2 X 10^6) were harvested and fixed with formaldehyde. Chromatin was fragmented by sonication to shear DNA to 200-500 bp in size. Chromatin fragments were immunoprecipitated using Dnmt3b antibody (1:50; Abcam, #ab2851). Histone H3 (1:50; Cell Signaling, #4620) and IgG (1:50; Cell Signaling, #2729) antibodies were used as positive and negative controls, respectively. After reverse crosslinking, the enrichment of *Rbpjk* DNA fragment was assessed by real-time qPCR. Primer sequences used for CpG islands in *Rbpjk* were forward, 5′-CTAGGTGACTCAGATGCATGAC-3′, and reverse, 5′-TCCCGAGCCTGGTACTT-3′; primer sequences for CpG Island 2 in *Rbpjk* gene body were forward, 5′-CCTTTGTTTCGCCGCCTTA-3′, and reverse, 5′-CCAGCTACTGAAGAGAGGGATA-3′.

Fabrication of cell impregnated tissue constructs. A simultaneous electrospinning and electrospraying approach was used to fabricate cell impregnated tissue constructs. The electrospraying stream contained suspension of adipose derived GFP+ progenitor cells (Cyagen, #MUBMD-01101) or dCas9-Dnmt3a-Rbpjk gRNA modified $C_3H_{10}T_{1/2}$ cells with a density of 4X10^6 cells/ml. The electrospinning stream was 10% polycaprolactone (PCL; Millipore Sigma, #440744) solution in chloroform. The electrospraying and electrospinning streams were loaded into syringe pumps. The needles of the two streams were charged at +18 kV and +24 kV, respectively. The injection rates of the PCL solution and cell suspension was controlled at 5 ml/h and 1.2 ml/h, respectively. The rotating collection mandrel was charged at -10 kV. The fabrication was conducted under an environment with 30% humidity. After fabricating for 30 minutes, the tissue construct was immersed in culture medium to remove residual solvent. The medium was changed twice. To observe live cells in the fabricated tissue constructs, confocal images were taken using a confocal microscope. Z-stack images were reconstructed. The morphology of tissue constructs was evaluated by SEM. To determine live cell density in the tissue constructs, the samples were incubated with papain solution followed by quantifying dsDNA content using Quant-iT dsDNA assay kit (ThermoFisher, #Q33233). The cell density was calculated based on calibration curve.

Supplemental figures

Figure S1. A schematic depicts experimental design for single cell RNA sequencing of fracture callus.

Figure S2. Systemic inflammation alters cellular distribution in fracture callus. (**A**) t-distributed stochastic embedding (tSNE) visualization of cell clustering in control and RA fracture callus. Annotated post hoc and colored by clustering. (**B**) Bar plots of inflammatory related cell counts in control and RA fracture callus.

Figure S3. Systemic inflammation alters genetic profiling of cells in fracture callus. Dot plots to visualize the changes of key feature expression across cell clusters. The size scale of the dots represents the percentage of cells within a cluster, while the color scale encodes the average expression level across all cells within a cluster. Key cell-type maker genes were plotted for (**A**) myeloid, (**B**) mesenchymal, and (**C**) endothelial lineage respectively. (**D**) Dot plots of key transcriptional factors of Notch, Wnt, TGFb, and Hedgehog signaling across cluster 13 and cluster 1 in control and RA fracture callus at 7 dpf.

Figure S4. RA, aging and obesity induces elevated inflammation in fracture callus. Real-time qPCR analyses of gene expression for *Il1b* and *Tnfa* in 5 dpf fracture callus isolated from (**A**) RA mice, (**B**) 18-month-old mice and (**C**) high-fat-diet induced obese mice (n=4). Data presented as mean ± SD. **p* < 0.05 by Student's t test.

Figure S5. IL1b treatment induces intrinsic inflammatory responses in primary PPCs. (**A**) Flow cytometry analysis of surface makers' expression in primary PPCs, including CD45, CD90 and Sca1. (**B**) Real-time qPCR analyses of gene expression for *Ikk2*, *Il1b*, *Il6*, and *Tnfa* in vehicle and IL1b treated primary PPCs isolated from 3-month-old wild type mice (n=3). Data presented as mean ± SD. **p* < 0.05 by Student's t test.

Figure S6. Cells in fracture callus are derived from Prx1-expressing cells. (**A**) Representative fluorescent images for tdTomato (Ai9) on callus sections from *Ai9*$^{f/+}$ and *Prx1Cre*^{ERT2}*;Ai9*$^{f/+}$ mice at 10 dpf. White dotted line outlined fracture callus. Scale bar, 100 μm. (**B**) Real-time qPCR analyses of gene expression for *Ikk2*, *Il1b* and *Tnfa* in 5 dpf callus tissue from *Ikk2ca*$^{f/+}$ (control) and *Prx1Cre*^{ERT2}*;Ikk2ca*$^{f/+}$ (*Ikk2ca*^{Prx1}) mice (n=5). Data presented as mean ± SD. **p* < 0.05 by Student's t test.

Figure S7. *Ikk2ca* in progenitor cells leads to reduced callus remodeling. (**A**) TRAP staining of 14 dpf fracture callus sections from *Ikk2ca*$^{f/+}$ (control) and *Prx1Cre*^{ERT2}*;Ikk2ca*$^{f/+}$ (*Ikk2ca*^{Prx1}) mice (n=5). Scale bar, 100 μm. (**B**) Oc.S/BS measured on TRAP staining of *Ikk2ca*$^{f/+}$ (control) and *Prx1Cre*^{ERT2}*;Ikk2ca*$^{f/+}$ (*Ikk2ca*^{Prx1}) fractures (n=5). Data presented as mean ± SD. **p* < 0.05 by Student's t test.

Figure S8. Histological assessments determine the effect of *Rbpjk* LOF on fracture repair defects induced by *Ikk2ca*. Histomorphometry analyses of mesenchyme, cartilage, and bone areas on callus sections from *Ikk2ca*$^{f/+}$ (control), *Prx1Cre*^{ERT2}*;Ikk2ca*$^{f/+}$ (*Ikk2ca*^{Prx1}), *Prx1Cre*^{ERT2}*;Rbpjk*$^{f/f}$ (*Rbpjk*^{Prx1}), and *Prx1Cre*^{ERT2}*;Ikk2ca*$^{f/+}$*;Rbpjk*$^{f/f}$ (*Ikk2ca;Rbpjk*^{Prx1}) mice at 7, 10, and 14 dpf (n=5). Data presented as mean ± SD. **p* < 0.05 by two-way ANOVA followed by Tukey test.

Figure S9. MicroCT assessments determine the effect of *Rbpjk* LOF on fracture repair defects induced by *Ikk2ca*. Quantifications of bony callus volume and BV/TV on reconstructed microCT images of *Ikk2ca*$^{f/+}$ (control), *Prx1Cre*^{ERT2}*;Ikk2ca*$^{f/+}$ (*Ikk2ca*^{Prx1}), *Prx1Cre*^{ERT2}*;Rbpjk*$^{f/f}$ (*Rbpjk*^{Prx1}), and *Prx1Cre*^{ERT2}*;Ikk2ca*$^{f/+}$*;Rbpjk*$^{f/f}$ (*Ikk2ca;Rbpjk*^{Prx1}) fractures at 14 dpf (n=5). Data presented as mean ± SD. **p* < 0.05 by two-way ANOVA followed by Tukey test.

Figure S10. TRAP staining assessments determine the effect of *Rbpjk* LOF on fracture repair defects induced by *Ikk2ca*. (**A**) TRAP staining of 14 dpf fracture callus sections from *Ikk2ca*$^{f/+}$ (control), *Prx1Cre*^{ERT2}*;Ikk2ca*$^{f/+}$ (*Ikk2ca*^{Prx1}), *Prx1Cre*^{ERT2}*;Rbpjk*$^{f/f}$ (*Rbpjk*^{Prx1}), and *Prx1Cre*^{ERT2}*;Ikk2ca*$^{f/+}$*;Rbpjk*$^{f/f}$ (*Ikk2ca;Rbpjk*^{Prx1}) mice (n=5). Scale bar, 100 μm. (**B**) Oc.S/BS measured on TRAP staining of *Ikk2ca*$^{f/+}$ (control), *Prx1Cre*^{ERT2}*;Ikk2ca*$^{f/+}$ (*Ikk2ca*^{Prx1}), *Prx1Cre*^{ERT2}*;Rbpjk*$^{f/f}$ (*Rbpjk*^{Prx1}), and *Prx1Cre*^{ERT2}*;Ikk2ca*$^{f/+}$*;Rbpjk*$^{f/f}$ (*Ikk2ca;Rbpjk*^{Prx1}) fractures (n=5). Data presented as mean ± SD. **p* < 0.05 by two-way ANOVA followed by Tukey test.

Figure S11. *Dnmt3b* expression is reduced in *Ikk2ca*-induced inflammatory callus. Real-time qPCR analyses of gene expression for *Dnmt1*, *Dnmt3a*, and *Dnmt3b* in 5 dpf callus tissue from *Ikk2ca*$^{f/+}$ (control) and *Prx1Cre*^{ERT2}*;Ikk2ca*$^{f/+}$ (*Ikk2ca*^{Prx1}) mice (n=5). Data presented as mean ± SD. **p* < 0.05 by Student's t test.

Figure S12. *Dnmt3b* is reduced in PPCs by inflammatory stimuli. Real-time qPCR analyses of gene expression for *Dnmt1*, *Dnmt3a*, and *Dnmt3b* in vehicle and IL1b treated primary wild type PPCs (n=3). Data presented as mean ± SD. **p* < 0.05 by Student's t test.

Figure S13. Rbpjk is a target of Dnmt3b. (**A**) Schematic illustration of the two Dnmt3b binding sites (CpG islands in green boxes) identified in *Rbpjk* gene. (**B**) Pull-down assay of genomic DNA with a Dnmt3b antibody (ChIP) showed interaction of Dnmt3b with its binding sites in *Rbpj*κ gene by qPCR (n=3). Data presented as mean ± SD. *$p < 0.05$ by two-way ANOVA followed by Tukey test.

Figure S14. DNA methylation reduction in *Rbpjk* gene leads to increased protein expression. (**A**) Schematic illustration of the two Dnmt3b binding sites (CpG islands in green boxes) identified in *Rbpjk* gene. Twelve gRNAs were designed to target CpG island1 (1.5 kb) and CpG island 2 (300 bp). (**B**) Schematic representing the selection of $C_3H_{10}T_{1/2}$ cell lines modified by dCas9-Tet1 epigenetic editing system specifically targeting CpG islands of *Rbpjk* gene. (**C**) Western blot analyses for Rbpjk in protein extracts from dCas9-Tet1-scramble gRNA (control) and 12 individual dCas9-Tet1-Rbpjk gRNAs engineered $C_3H_{10}T_{1/2}$ cell lines (n=3). C, dCas9-Tet1-scramble gRNA. 1-10, dCas9-Tet1-$Rbpjk_{1\text{-}1\text{-}10}$ gRNAs targeting CpG island 1 of *Rbpjk* gene. 11-12, dCas9-Tet1-$Rbpjk_{2\text{-}1\text{-}2}$. gRNAs targeting CpG island 2 of *Rbpjk* gene.

Figure S15. Gene expression of progenitor differentiation markers to determine the effect of Rbpjk inhibition using an epigenetic modification approach on progenitor differentiation potential. Real-time qPCR analyses for *Rbpjk*, *Hey1*, *Sox9*, *Col2a1*, *Acan*, *Sp7*, *Rux2*, and *Alp* in dCas9-Dnmt3a-scramble (control) and dCas9-Dnmt3a-$Rbpjk_{1\text{-}9}$ $C_3H_{10}T_{1/2}$ (*Rbpjk* epigenetically modified) cell lines in the presence or absence of IL1b. Data presented as mean ± SD. *$p < 0.05$ by two-way ANOVA followed by Tukey test.

Figure S16. Tamoxifen and Doxycycline induces *Ikk2* and *Dnmt3b* gene expression in fracture callus. Real-time qPCR analyses for *Ikk2* and *Dnmt3b* in 5 dpf callus from *Ikk2ca*$^{f/+}$ (control) and *Prx1Cre*^{ERT2}*;Ikk2ca*$^{f/+}$*;Rosa-rtTA*$^{f/+}$*;Dnmt3b-tg* (*Ikk2ca;Dnmt3b-tg*^{Prx1}) mice (n=5). Data presented as mean ± SD. *$p < 0.05$ by Student's t test.

Figure S17. Histological analyses determine the effect of *Dnmt3b* GOF on fracture repair defects induced by *Ikk2ca*. Histomorphometry quantifications of mesenchyme and cartilage areas on callus sections from *Ikk2ca*$^{f/+}$ (control), *Prx1Cre*^{ERT2}*;Ikk2ca*$^{f/+}$ (*Ikk2ca*^{Prx1}), *Prx1Cre*^{ERT2}*;Rosa-rtTA*$^{f/+}$*;Dnmt3b-tg* (*Dnmt3b-tg*^{Prx1}), and *Prx1Cre*^{ERT2}*;Ikk2ca*$^{f/+}$*;Rosa-rtTA*$^{f/+}$*;Dnmt3b-tg* (*Ikk2ca;Dnmt3b-tg*^{Prx1}) mice at 7 and 10dpf, respectively (n=5). Data presented as mean ± SD. *$p < 0.05$ by two-way ANOVA followed by Tukey test.

Figure S18. MicroCT analyses determine the effect of *Dnmt3b* GOF on fracture repair defects induced by *Ikk2ca*. (**A**) MicroCT images of mineralized bony calluses from *Ikk2ca*$^{f/+}$ (control), *Prx1Cre*^{ERT2}*;Ikk2ca*$^{f/+}$ (*Ikk2ca*^{Prx1}), *Prx1Cre*^{ERT2}*;Rosa-rtTA*$^{f/+}$*;Dnmt3b-tg* (*Dnmt3b-tg*^{Prx1}), and *Prx1Cre*^{ERT2}*;Ikk2ca*$^{f/+}$*;Rosa-rtTA*$^{f/+}$*;Dnmt3b-tg* (*Ikk2ca;Dnmt3b-tg*^{Prx1}) fractures at 14 dpf (n=5). Scale bar, 0.5 mm. (**B**) Quantifications of bony callus volume and BV/TV on reconstructed microCT images of *Ikk2ca*$^{f/+}$ (control), *Prx1Cre*^{ERT2}*;Ikk2ca*$^{f/+}$ (*Ikk2ca*^{Prx1}), *Prx1Cre*^{ERT2}*;Rosa-rtTA*$^{f/+}$*;Dnmt3b-tg* (*Dnmt3b-tg*^{Prx1}), and *Prx1Cre*^{ERT2}*;Ikk2ca*$^{f/+}$*;Rosa-rtTA*$^{f/+}$*;Dnmt3b-tg* (*Ikk2ca;Dnmt3b-tg*^{Prx1}) fractures at 14 dpf (n=5). Data presented as mean ± SD. *$p < 0.05$ by two-way ANOVA followed by Tukey test.

Figure S19. *Rbpjk* inhibition restores *Dnmt3b* deficiency mediated PPC differentiation defects. Chondrogenic pellet and osteogenic differentiation assays were performed within *Dnmt3b*$^{f/f}$ PPCs following Ad-GFP (control) or Ad-Cre (*Dnmt3b* LOF) transfection in the presence or absence of *Rbpjk* LOF. Alcian blue and alizarin red staining of chondrogenic pellet sections and osteogenic cultures at day 28 and day 21, respectively (n=3).

Figure S20. Histological analyses determine the effect of *Rbpjk* LOF on fracture repair defects induced by *Dnmt3b* LOF. Histomorphometry quantifications of mesenchyme and cartilage areas on callus sections from *Dnmt3b*$^{f/f}$ (control), *Prx1Cre*^{ERT2}*;Dnmt3b*$^{f/f}$ (*Dnmt3b*^{Prx1}),

Prx1Cre^{ERT2}*;Rbpjk*$^{f/f}$ (*Rbpjk*^{Prx1}), and *Prx1Cre*^{ERT2}*; Dnmt3b*$^{f/f}$*;Rbpjk*$^{f/f}$ (*Dnmt3b;Rbpjk*^{Prx1}) mice at 7 and 10dpf, respectively (n=5). Data presented as mean ± SD. *$p < 0.05$ by two-way ANOVA followed by Tukey test.

Figure S21. MicroCT analyses determine the effect of *Rbpjk* LOF on fracture repair defects induced by *Dnmt3b* LOF. (**A**) MicroCT images of mineralized bony calluses from *Dnmt3b*$^{f/f}$ (control), *Prx1Cre*^{ERT2}*;Dnmt3b*$^{f/f}$ (*Dnmt3b*^{Prx1}), *Prx1Cre*^{ERT2}*;Rbpjk*$^{f/f}$ (*Rbpjk*^{Prx1}), and *Prx1Cre*^{ERT2}*; Dnmt3b*$^{f/f}$*;Rbpjk*$^{f/f}$ (*Dnmt3b;Rbpjk*^{Prx1}) fractures at 14 dpf (n=5). Scale bar, 0.5 mm. (**B**) Quantifications of bony callus volume and BV/TV on reconstructed microCT images of *Dnmt3b*$^{f/f}$ (control), *Prx1Cre*^{ERT2}*;Dnmt3b*$^{f/f}$ (*Dnmt3b*^{Prx1}), *Prx1Cre*^{ERT2}*;Rbpjk*$^{f/f}$ (*Rbpjk*^{Prx1}), and *Prx1Cre*^{ERT2}*; Dnmt3b*$^{f/f}$*;Rbpjk*$^{f/f}$ (*Dnmt3b;Rbpjk*^{Prx1}) fractures at 14 dpf (n=5). Data presented as mean ± SD. *$p < 0.05$ by two-way ANOVA followed by Tukey test.

Figure S22. TRAP staining assessments determine the effect of *Rbpjk* LOF on RA fracture repair. (**A**) TRAP staining of 14 dpf fracture callus sections from *Rbpjk*$^{f/f}$ (control) and *Prx1Cre*^{ERT2}*;Rbpjk*$^{f/f}$ (*Rbpjk*^{Prx1}) RA mice (n=5). Scale bar, 100 μm. (**B**) Oc.S/BS measured on TRAP staining of *Rbpjk*$^{f/f}$ (control) and *Prx1Cre*^{ERT2}*;Rbpjk*$^{f/f}$ (*Rbpjk*^{Prx1}) RA fractures (n=5). Data presented as mean ± SD. *$p < 0.05$ by Student's t test.

Figure S23. Progenitors fabricated in PCL scaffolds are capable of differentiating and forming calluses at fracture site. (**A**) Schematic illustration of the simultaneous electrospinning and electrospraying system for cell impregnated tissue construct fabrication. (**B**) Representative SEM images of the PCL scaffold with adipose derived GFP+ progenitor cells (n=3). (**C**) Representative confocal images of PCL scaffolds with adipose derived GFP+ progenitor cells (n=3). (**D**) Fluorescent images of frozen callus sections of 10-dpf wild type fractures grafted with PCL scaffolds that were fabricated with adipose derived GFP+ progenitor cells. White dotted line outlined cortical bones. Scale bar, 100 μm.

Figure S24. Angiogenesis is restored by epigenetically modified cells on RA fracture callus. Immunofluorescent staining for endomucin in 14 dpf callus sections from wild type RA fractures grafted with PCL scaffolds that were fabricated with dCas9-Dnmt3a-scramble (control) or dCas9-Dnmt3a-Rbpjk$_{1-9}$ $C_3H_{10}T_{1/2}$ cells (*Rbpjk* epigenetically modified) (n=5). Scale bar, 100 μm.

Figure S25. TRAP staining assessments to determine the effect of epigenetically modified cells on RA fracture repair. (**A**) TRAP staining of callus sections from 14 dpf wild type RA fractures grafted with PCL scaffolds that were fabricated with dCas9-Dnmt3a-scramble (control) or dCas9-Dnmt3a-Rbpjk$_{1-9}$ $C_3H_{10}T_{1/2}$ cells (*Rbpjk* epigenetically modified) (n=5). Scale bar, 100 μm. (**B**) Oc.S/BS measured on TRAP staining of RA fractures grafted with PCL scaffolds that were fabricated with dCas9-Dnmt3a-scramble (control) or dCas9-Dnmt3a-Rbpjk$_{1-9}$ $C_3H_{10}T_{1/2}$ cells (*Rbpjk* epigenetically modified) (n=5). Data presented as mean ± SD. *$p < 0.05$ by Student's t test.

Fig. S1

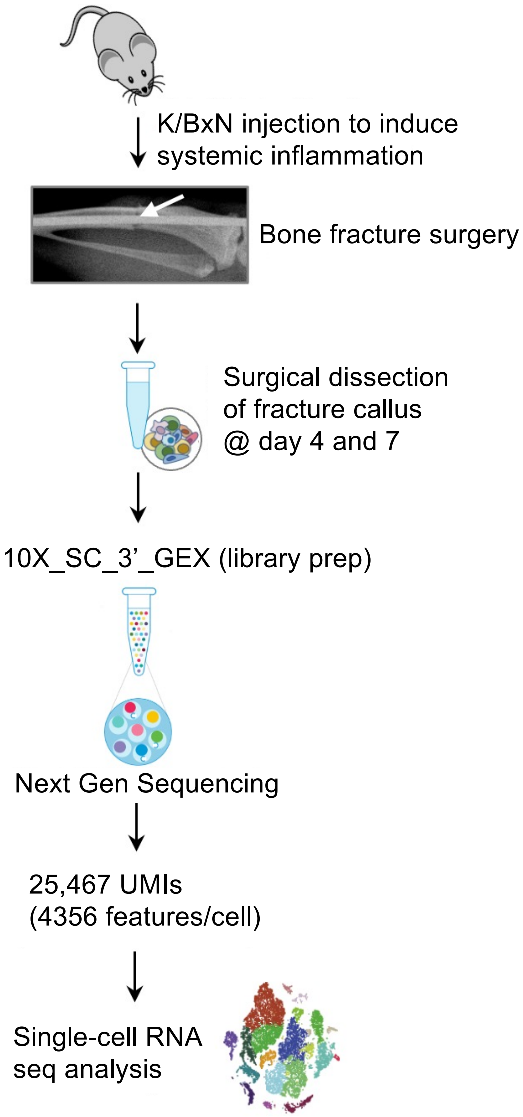
K/BxN injection to induce
systemic inflammation
Bone fracture surgery
Surgical dissection
of fracture callus
@ day 4 and 7
10X_SC_3'_GEX (library prep)
Next Gen Sequencing
25,467 UMIs
(4356 features/cell)
Single-cell RNA
seq analysis

Fig. S2

A

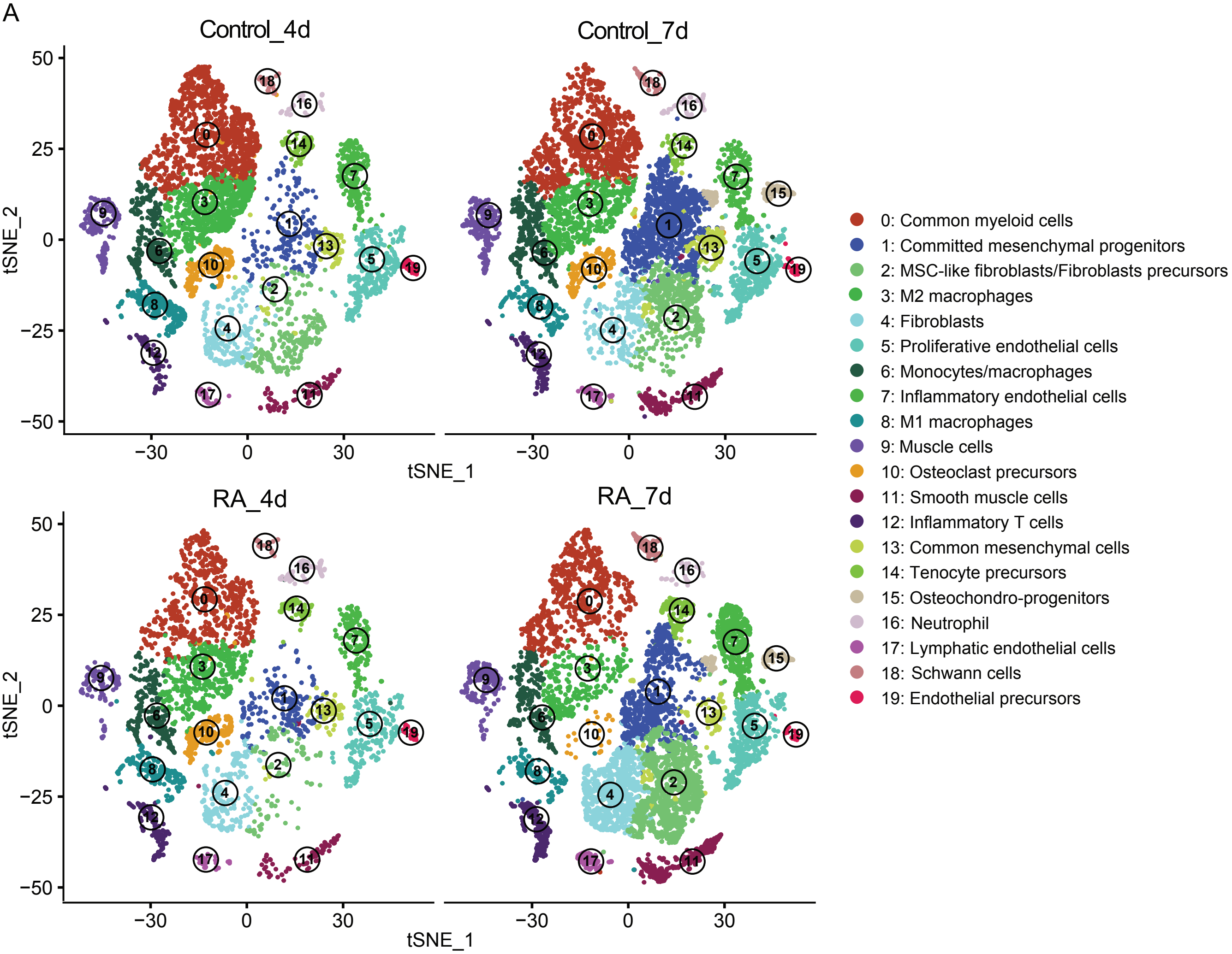
Control_4d
Control_7d
RA_4d
RA_7d
tSNE_1
tSNE_2
0: Common myeloid cells
1: Committed mesenchymal progenitors
2: MSC-like fibroblasts/Fibroblasts precursors
3: M2 macrophages
4: Fibroblasts
5: Proliferative endothelial cells
6: Monocytes/macrophages
7: Inflammatory endothelial cells
8: M1 macrophages
9: Muscle cells
10: Osteoclast precursors
11: Smooth muscle cells
12: Inflammatory T cells
13: Common mesenchymal cells
14: Tenocyte precursors
15: Osteochondro-progenitors
16: Neutrophil
17: Lymphatic endothelial cells
18: Schwann cells
19: Endothelial precursors

B

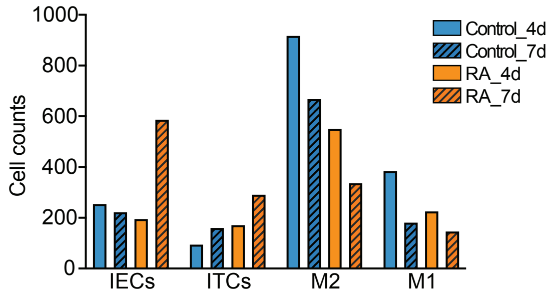
Cell counts
1000
800
600
400
200
0
IECs
ITCs
M2
M1
Control_4d
Control_7d
RA_4d
RA_7d

Fig. S3

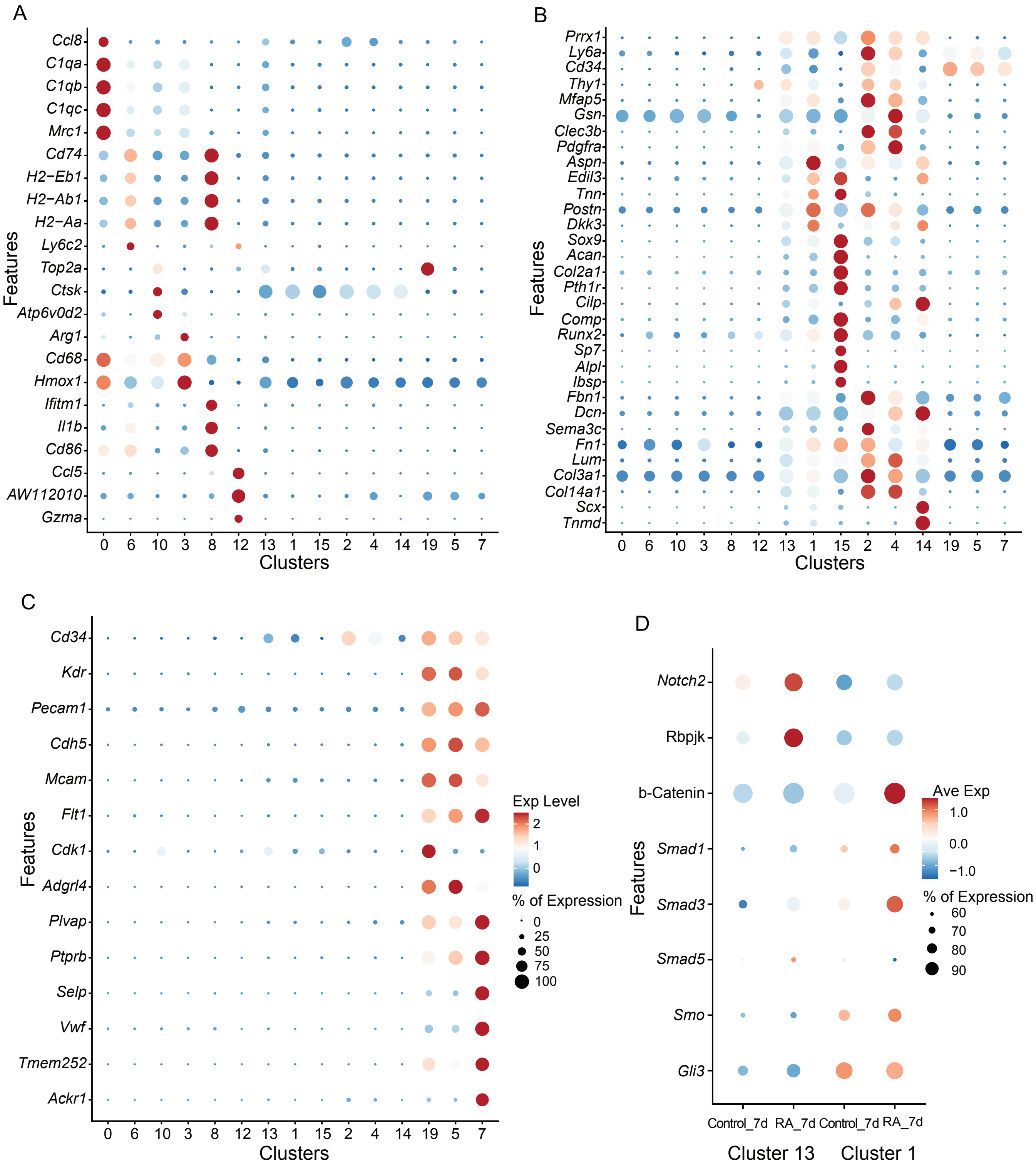
A
Ccl8
C1qa
C1qb
C1qc
Mrc1
Cd74
H2−Eb1
H2−Ab1
H2−Aa
Ly6c2
Top2a
Ctsk
Atp6v0d2
Arg1
Cd68
Hmox1
Ifitm1
Il1b
Cd86
Ccl5
AW112010
Gzma
Features
0 6 10 3 8 12 13 1 15 2 4 14 19 5 7
Clusters
B
Prrx1
Ly6a
Cd34
Thy1
Mfap5
Gsn
Clec3b
Pdgfra
Aspn
Edil3
Tnn
Postn
Dkk3
Sox9
Acan
Col2a1
Pth1r
Cilp
Comp
Runx2
Sp7
Alpl
Ibsp
Fbn1
Dcn
Sema3c
Fn1
Lum
Col3a1
Col14a1
Scx
Tnmd
Features
0 6 10 3 8 12 13 1 15 2 4 14 19 5 7
Clusters
C
Cd34
Kdr
Pecam1
Cdh5
Mcam
Flt1
Cdk1
Adgrl4
Plvap
Ptprb
Selp
Vwf
Tmem252
Ackr1
Features
0 6 10 3 8 12 13 1 15 2 4 14 19 5 7
Clusters
Exp Level
2
1
0
% of Expression
0
25
50
75
100
D
Notch2
Rbpjk
b-Catenin
Smad1
Smad3
Smad5
Smo
Gli3
Features
Control_7d RA_7d Control_7d RA_7d
Cluster 13 Cluster 1
Ave Exp
1.0
0.0
−1.0
% of Expression
60
70
80
90

Fig. S4

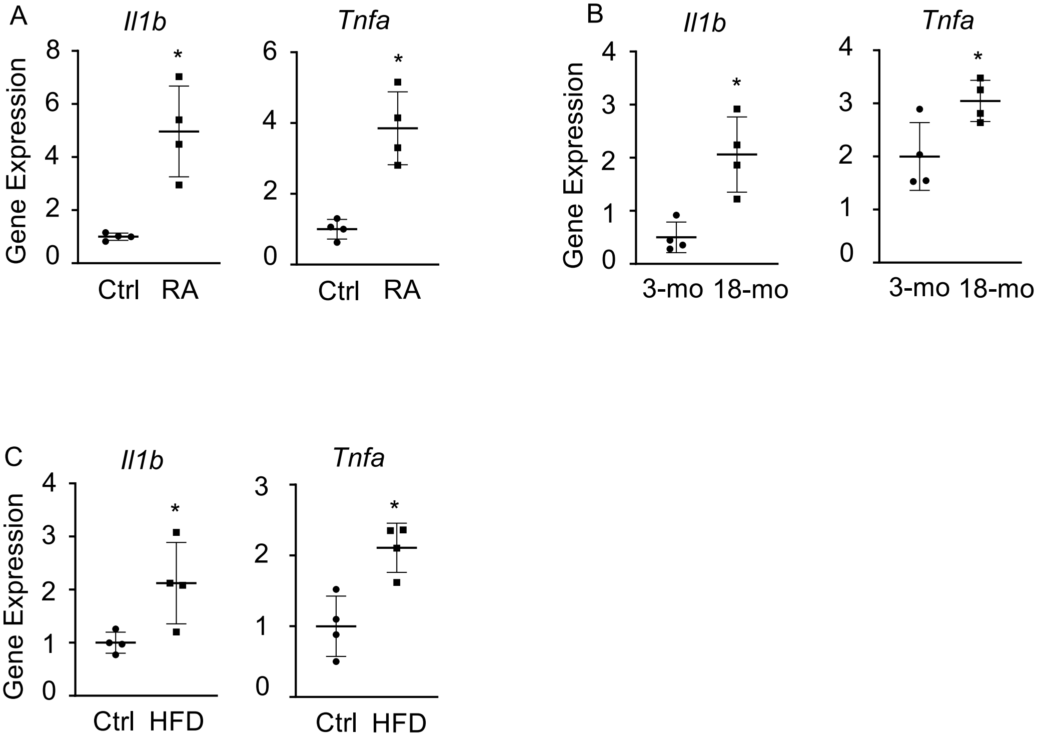

Fig. S5

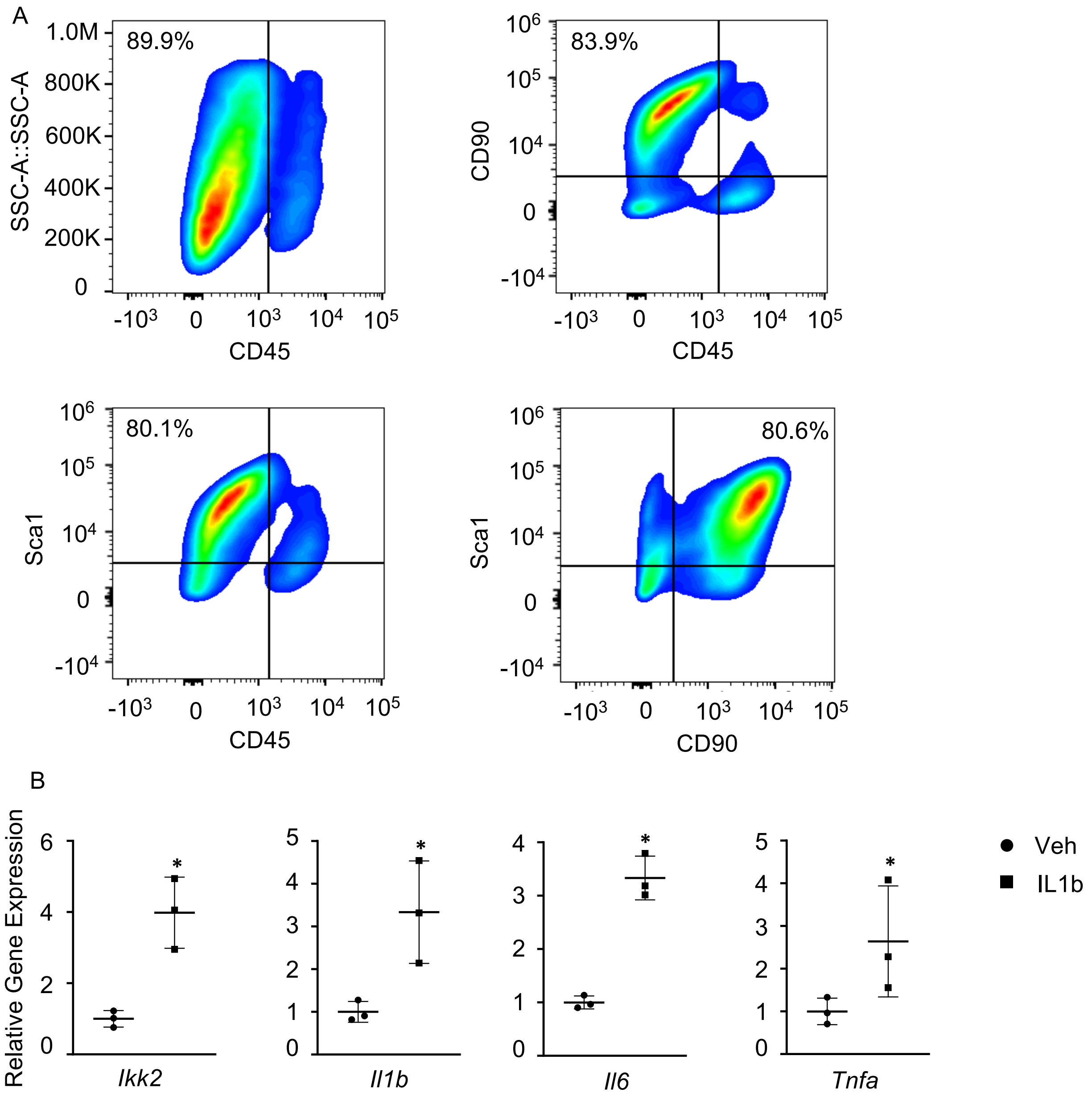
A
89.9%
SSC-A::SSC-A
CD45
83.9%
CD90
CD45
80.1%
Sca1
CD45
80.6%
Sca1
CD90
B
Relative Gene Expression
Ikk2
Il1b
Il6
Tnfa
Veh
IL1b

Fig. S6

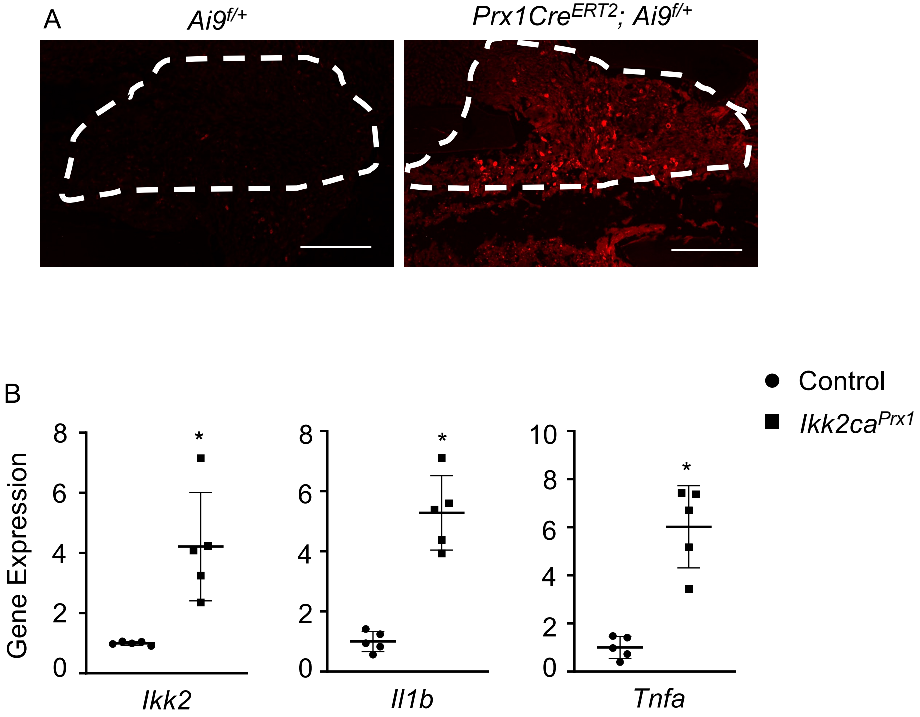
A
Ai9f/+
Prx1CreERT2; Ai9f/+
B
Control
Ikk2caPrx1
Gene Expression
Ikk2
Il1b
Tnfa
*

Fig. S7

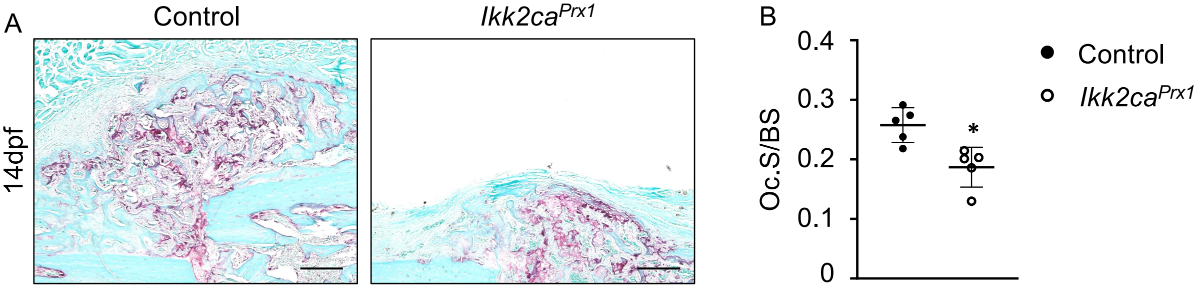
A
Control
Ikk2ca^Prx1
14dpf
B
0.4
0.3
0.2
0.1
0
Oc.S/BS
*
Control
Ikk2ca^Prx1

Fig. S8

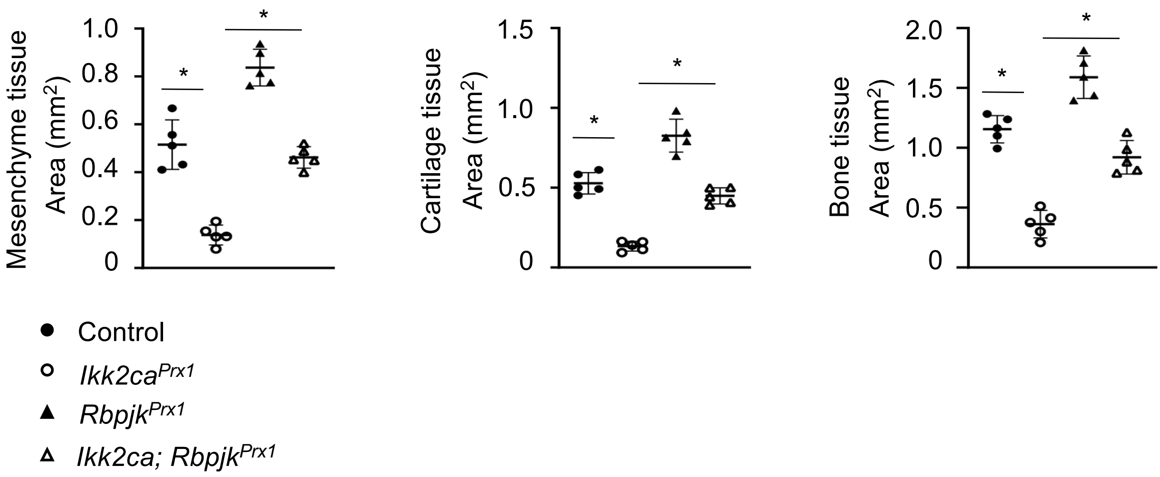

Mesenchyme tissue
Area (mm2)
1.0
0.8
0.6
0.4
0.2
0
*
*
Cartilage tissue
Area (mm2)
1.5
1.0
0.5
0
*
*
Bone tissue
Area (mm2)
2.0
1.5
1.0
0.5
0
*
*
Control
Ikk2ca^Prx1
Rbpjk^Prx1
Ikk2ca; Rbpjk^Prx1

Fig. S9

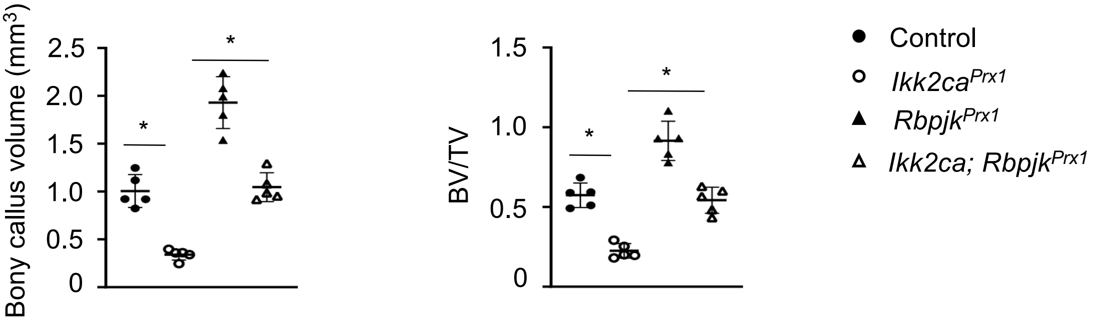

Bony callus volume (mm3)
2.5
2.0
1.5
1.0
0.5
0
*
*
BV/TV
1.5
1.0
0.5
0
*
*
Control
Ikk2caPrx1
RbpjkPrx1
Ikk2ca; RbpjkPrx1

Fig. S10

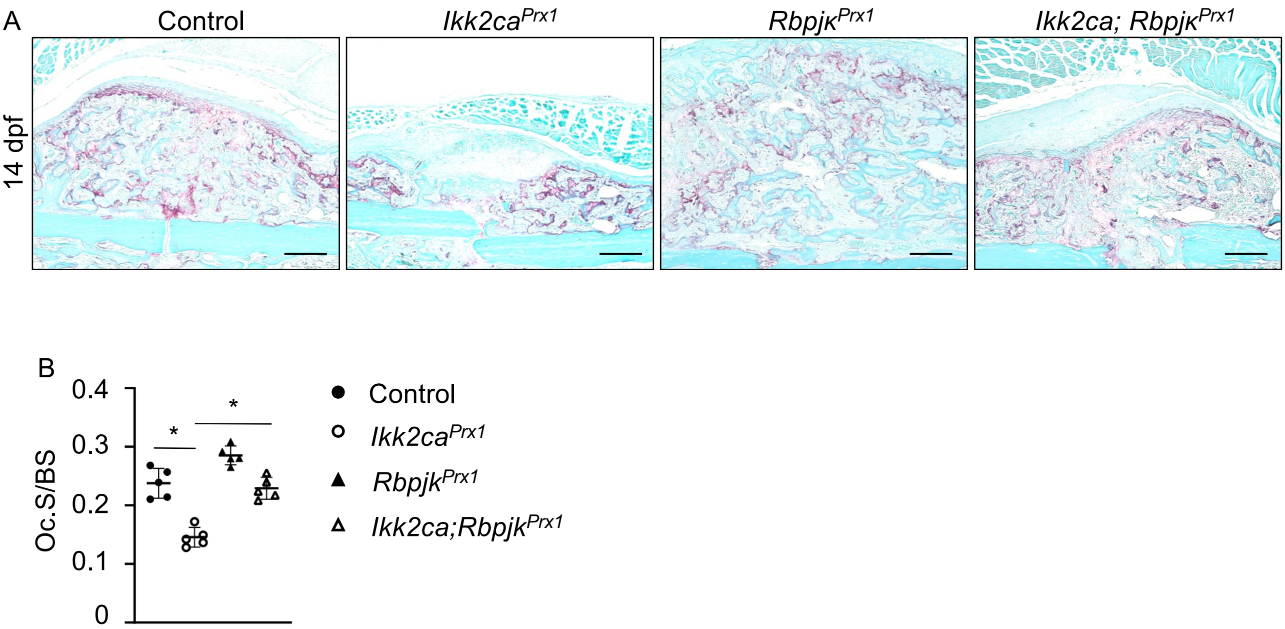

A
Control
Ikk2ca^{Prx1}
Rbpjκ^{Prx1}
Ikk2ca; Rbpjκ^{Prx1}
14 dpf
B
Oc.S/BS
0.4
0.3
0.2
0.1
0
*
*
Control
Ikk2ca^{Prx1}
Rbpjk^{Prx1}
Ikk2ca;Rbpjk^{Prx1}

Fig. S11

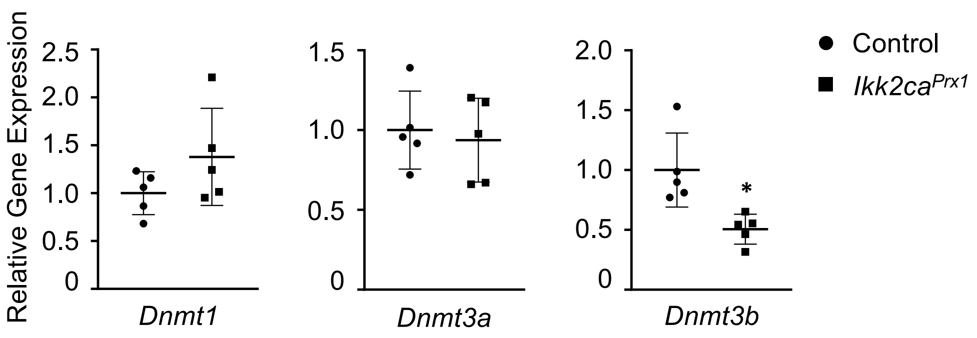

Relative Gene Expression
2.5
2.0
1.5
1.0
0.5
0
Dnmt1
1.5
1.0
0.5
0
Dnmt3a
2.0
1.5
1.0
0.5
0
*
Dnmt3b
Control
Ikk2ca^Prx1

Fig. S12

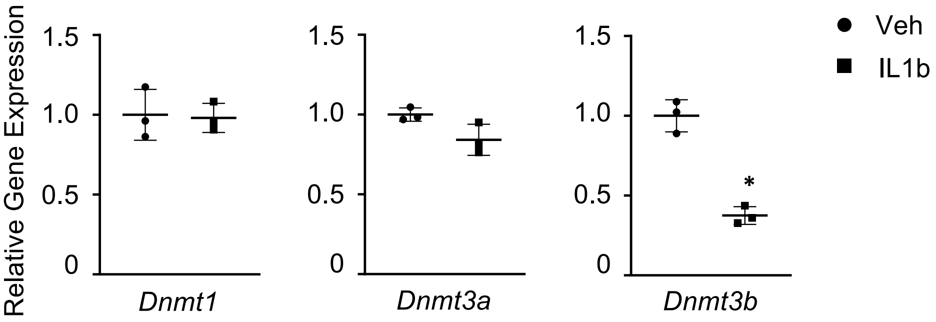
Relative Gene Expression
1.5
1.0
0.5
0
Dnmt1
Dnmt3a
Dnmt3b
*
Veh
IL1b

Fig. S13

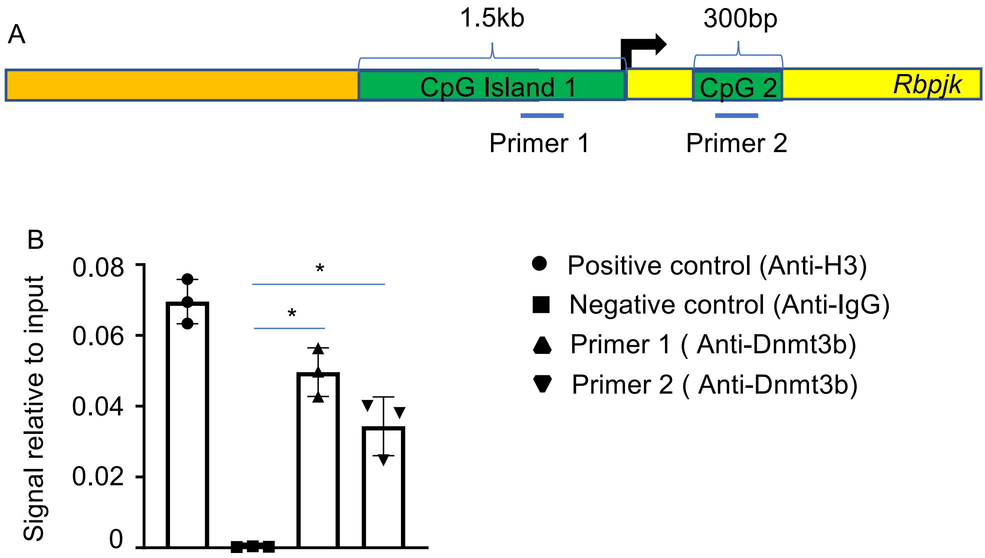

A
1.5kb
300bp
CpG Island 1
CpG 2
Rbpjk
Primer 1
Primer 2
B
Signal relative to input
0.08
0.06
0.04
0.02
0
*
*
Positive control (Anti-H3)
Negative control (Anti-IgG)
Primer 1 (Anti-Dnmt3b)
Primer 2 (Anti-Dnmt3b)

Fig. S14

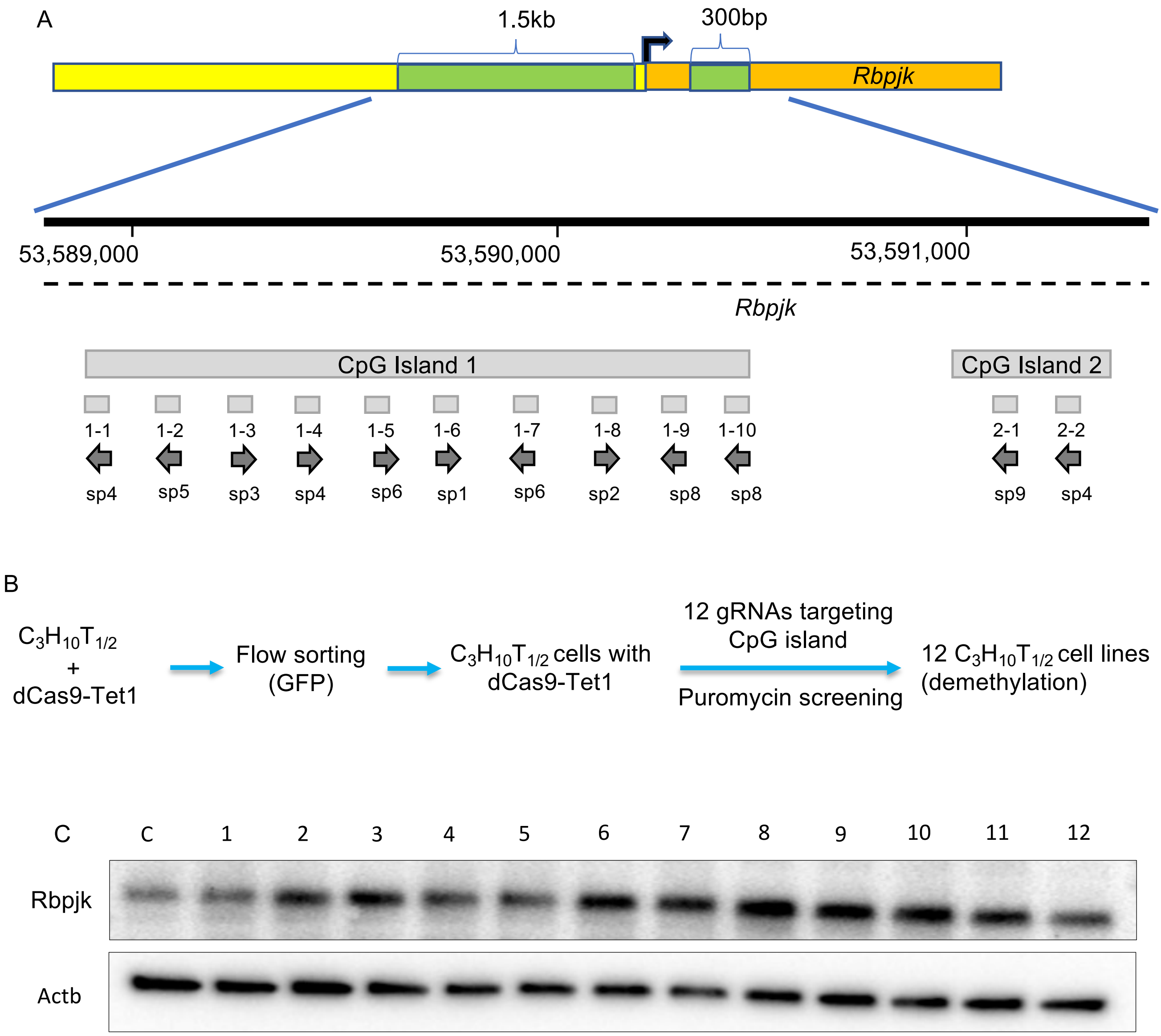
A
1.5kb
300bp
Rbpjk
53,589,000
53,590,000
53,591,000
Rbpjk
CpG Island 1
CpG Island 2
1-1
1-2
1-3
1-4
1-5
1-6
1-7
1-8
1-9
1-10
2-1
2-2
sp4
sp5
sp3
sp4
sp6
sp1
sp6
sp2
sp8
sp8
sp9
sp4
B
$C_3H_{10}T_{1/2}$ + dCas9-Tet1
Flow sorting (GFP)
$C_3H_{10}T_{1/2}$ cells with dCas9-Tet1
12 gRNAs targeting CpG island
Puromycin screening
12 $C_3H_{10}T_{1/2}$ cell lines (demethylation)
C
C
1
2
3
4
5
6
7
8
9
10
11
12
Rbpjk
Actb

Fig. S15

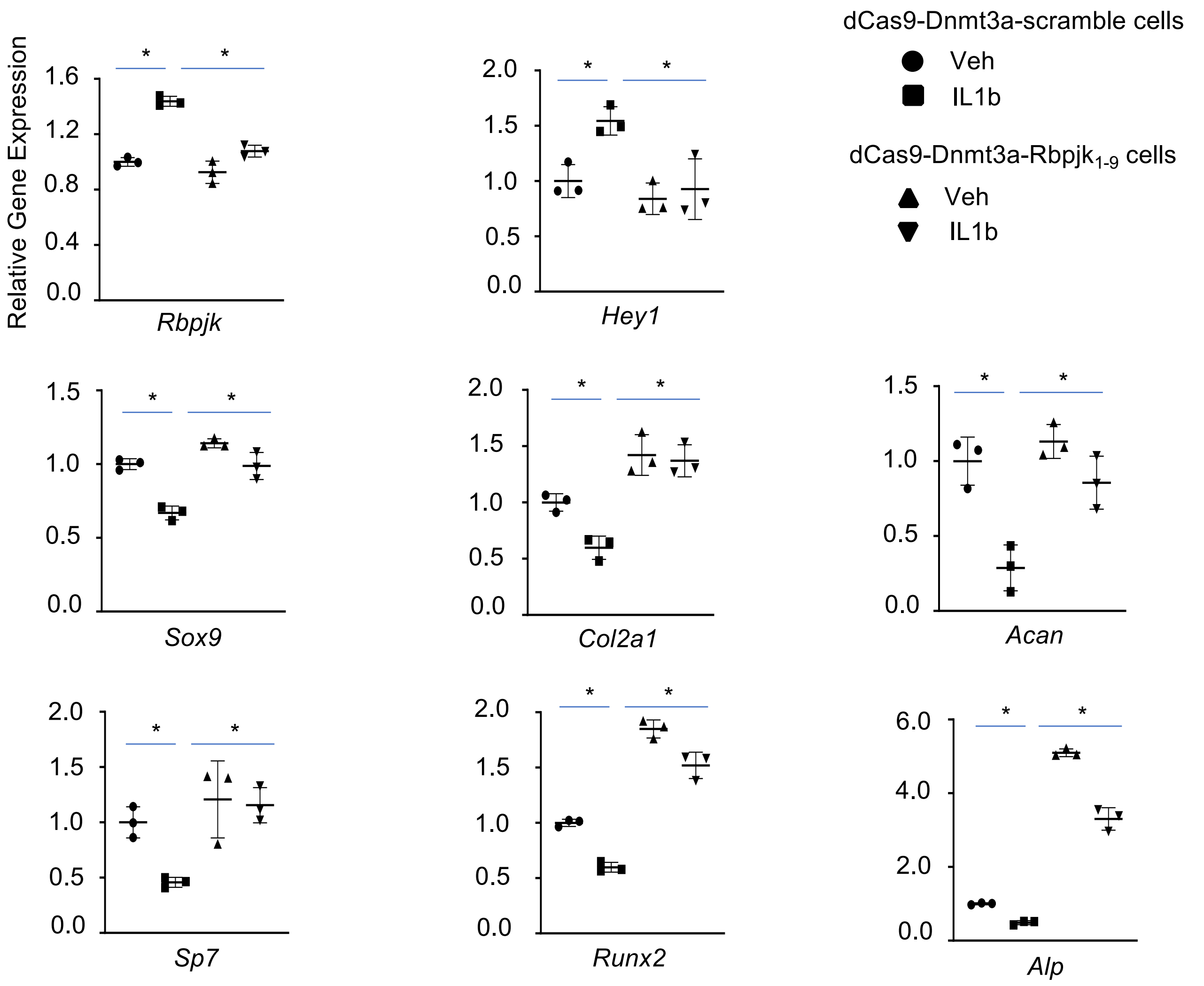

dCas9-Dnmt3a-scramble cells
Veh
IL1b
dCas9-Dnmt3a-Rbpjk$_{1-9}$ cells
Veh
IL1b
Relative Gene Expression
Rbpjk
Hey1
Sox9
Col2a1
Acan
Sp7
Runx2
Alp

Fig. S16

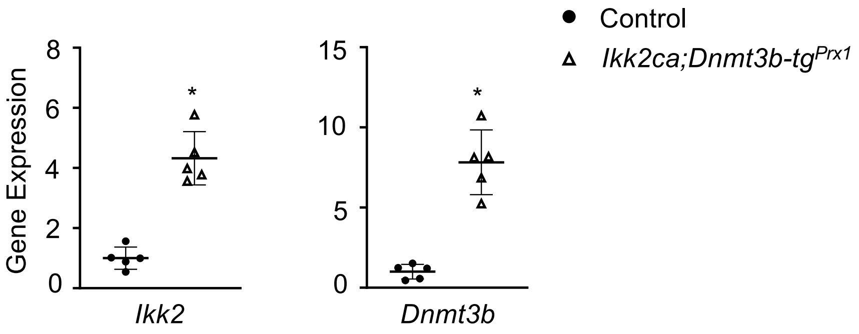
Control
Ikk2ca;Dnmt3b-tgPrx1
Gene Expression
8
6
4
2
0
*
Ikk2
15
10
5
0
*
Dnmt3b

Fig. S17

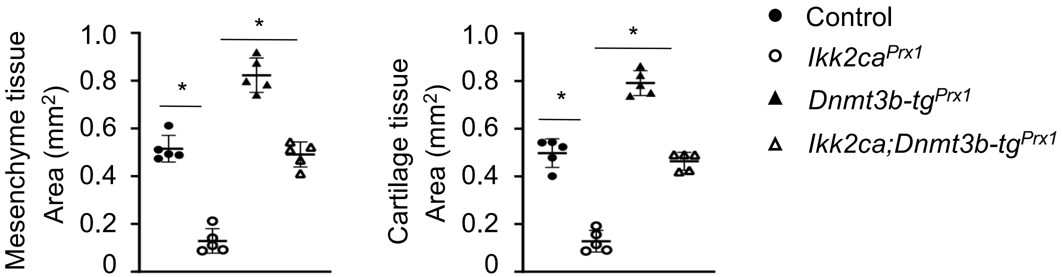

Mesenchyme tissue Area (mm2)
1.0
0.8
0.6
0.4
0.2
0
*
*
Cartilage tissue Area (mm2)
1.0
0.8
0.6
0.4
0.2
0
*
*
Control
Ikk2caPrx1
Dnmt3b-tgPrx1
Ikk2ca;Dnmt3b-tgPrx1

Fig. S18

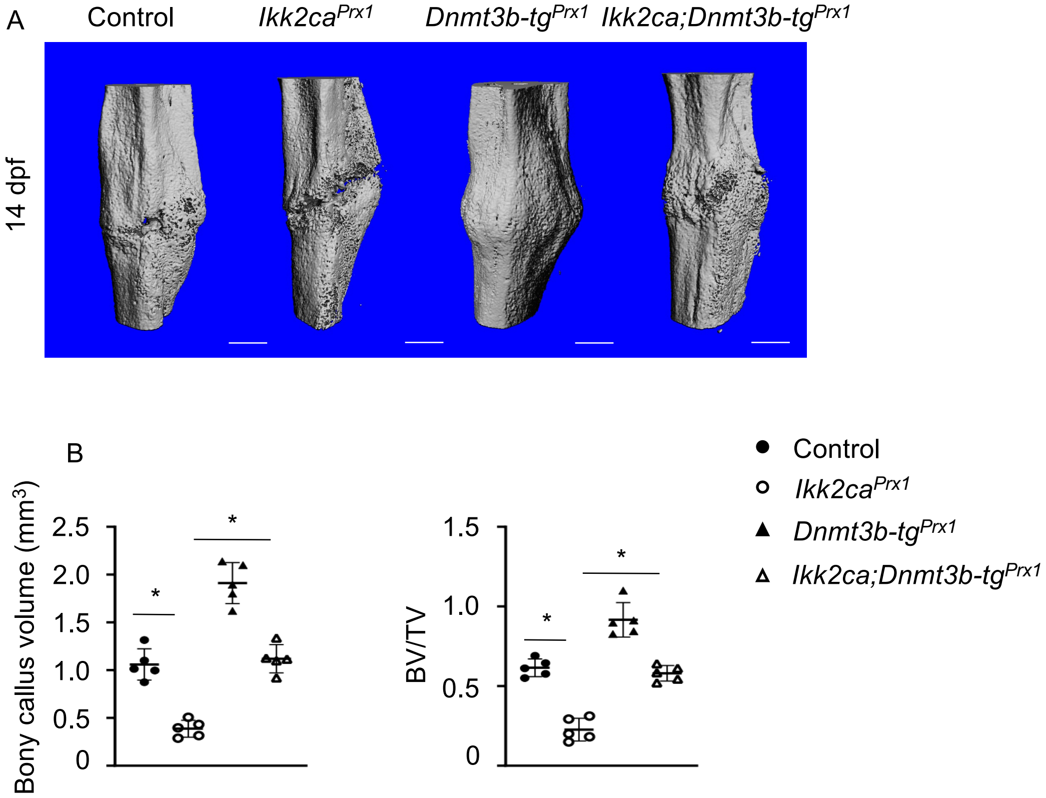
A
Control
Ikk2caPrx1
Dnmt3b-tgPrx1
Ikk2ca;Dnmt3b-tgPrx1
14 dpf
B
Bony callus volume (mm3)
2.5
2.0
1.5
1.0
0.5
0
*
*
BV/TV
1.5
1.0
0.5
0
*
*
Control
Ikk2caPrx1
Dnmt3b-tgPrx1
Ikk2ca;Dnmt3b-tgPrx1

Fig. S19

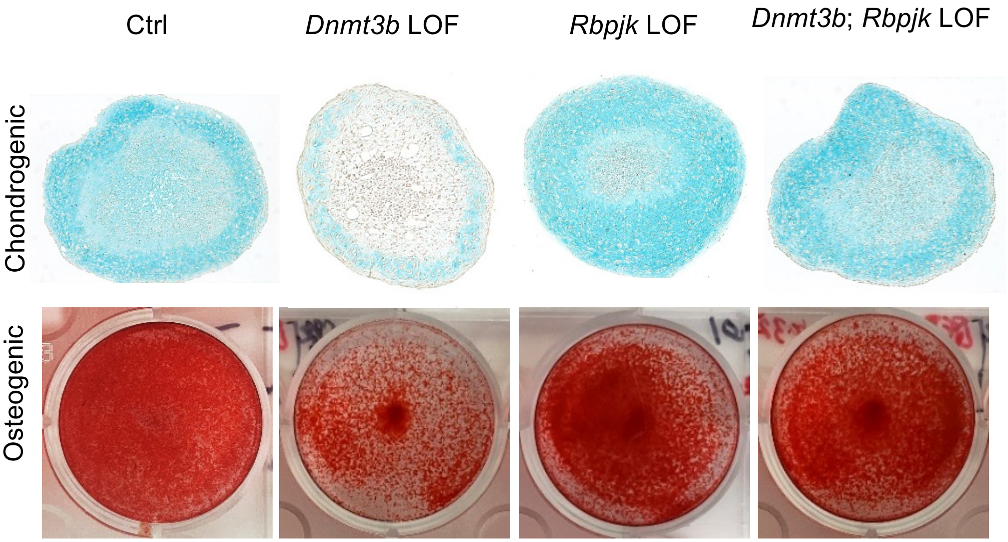

Ctrl
Dnmt3b LOF
Rbpjk LOF
Dnmt3b; *Rbpjk* LOF
Chondrogenic
Osteogenic

Fig. S20

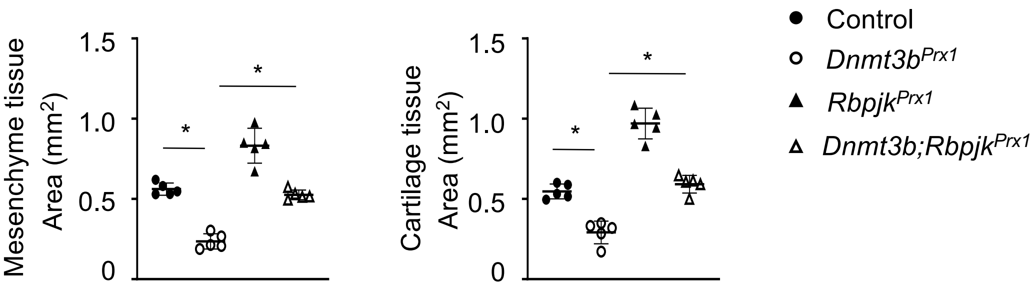
Mesenchyme tissue
Area (mm2)
1.5
1.0
0.5
0
*
*
Cartilage tissue
Area (mm2)
1.5
1.0
0.5
0
*
*
Control
Dnmt3bPrx1
RbpjkPrx1
Dnmt3b;RbpjkPrx1

Fig. S21

A

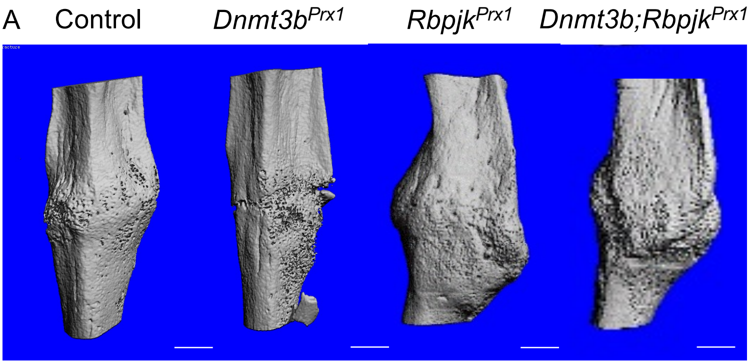

Control
Dnmt3b^{Prx1}
Rbpjk^{Prx1}
Dnmt3b;Rbpjk^{Prx1}

B

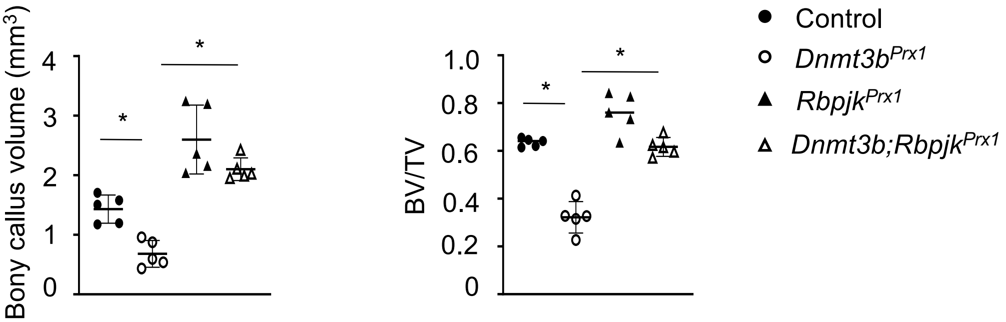

Bony callus volume (mm^3)
4
3
2
1
0
*
*
BV/TV
1.0
0.8
0.6
0.4
0.2
0
*
*
Control
Dnmt3b^{Prx1}
Rbpjk^{Prx1}
Dnmt3b;Rbpjk^{Prx1}

Fig. S22

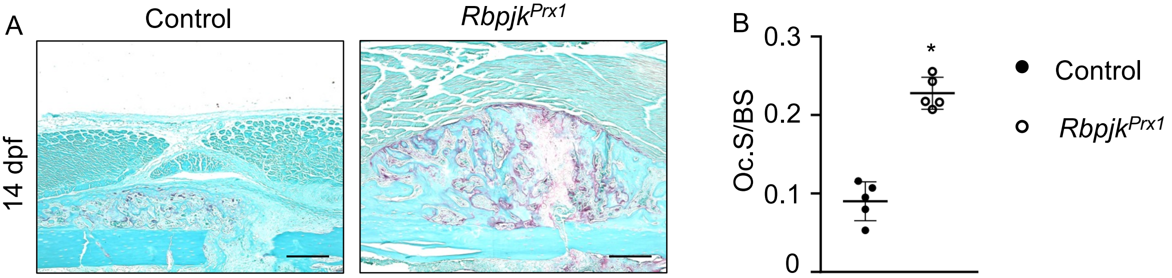
A
Control
$Rbpjk^{Prx1}$
14 dpf
B
Oc.S/BS
0.3
0.2
0.1
0
*
Control
$Rbpjk^{Prx1}$

Fig. S23

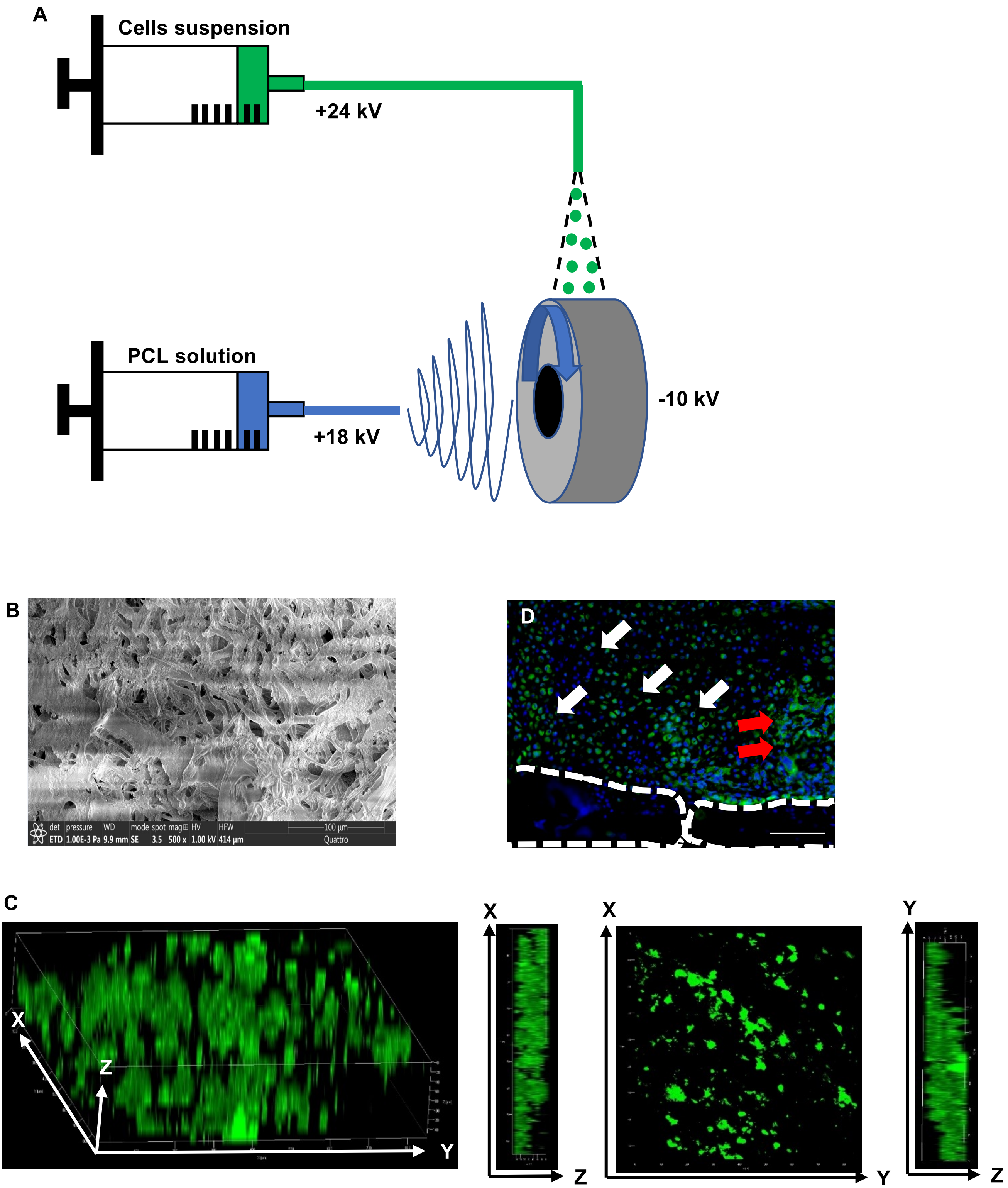
A
Cells suspension
+24 kV
PCL solution
+18 kV
-10 kV
B
det pressure WD mode spot mag HV HFW
ETD 1.00E-3 Pa 9.9 mm SE 3.5 500 x 1.00 kV 414 μm
100 μm
Quattro
D
C
X
Z
Y
X
Z
X
Y
Y
Z

Fig. S24

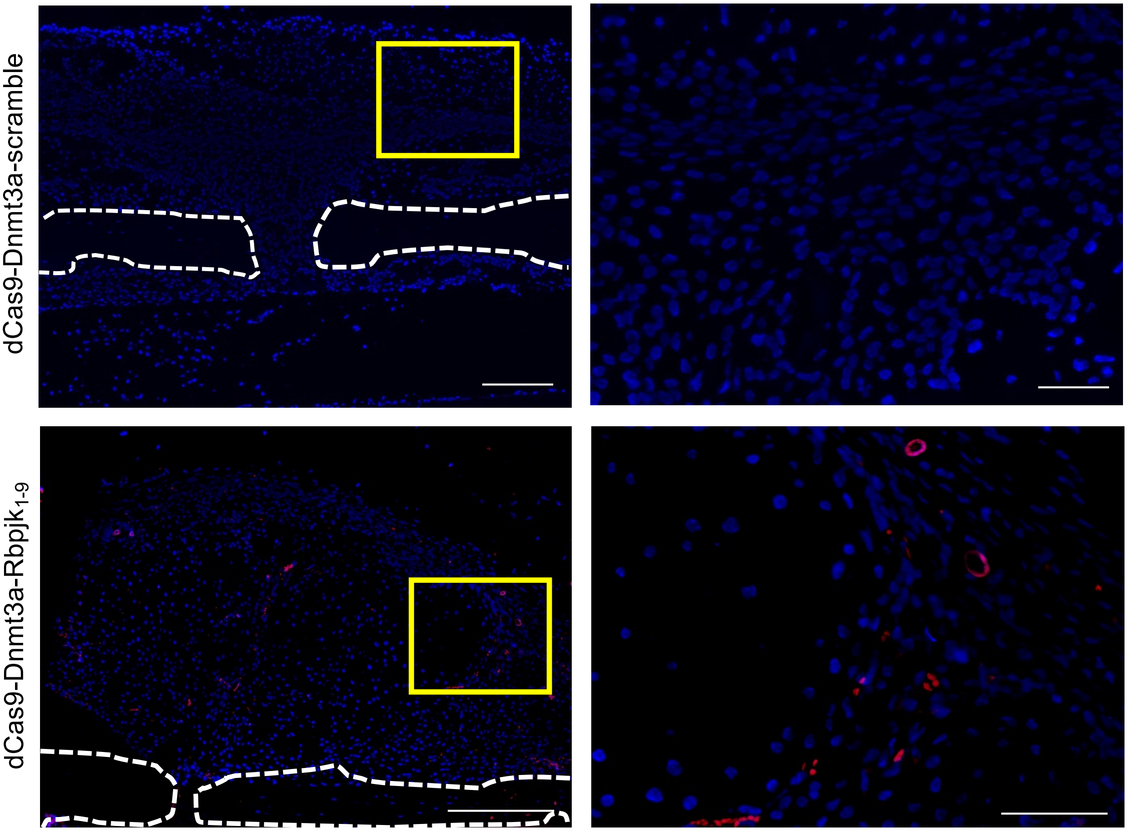

Fig. S25

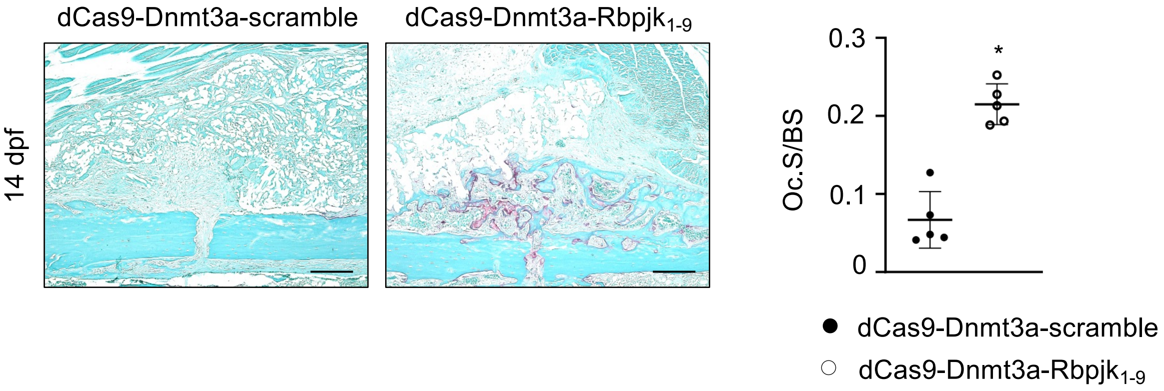

dCas9-Dnmt3a-scramble
dCas9-Dnmt3a-Rbpjk1-9
14 dpf
Oc.S/BS
0.3
0.2
0.1
0
*
dCas9-Dnmt3a-scramble
dCas9-Dnmt3a-Rbpjk1-9
